# Crystals as Rockets: Modulation of the Salient Temperature in Cocrystals by Solvent Mixture Composition

**DOI:** 10.1002/anie.202526010

**Published:** 2026-03-18

**Authors:** Ernesto A. Hernández‐Morales, Dayra Barreto‐Hernández, Dazaet Galicia‐Badillo, Rubén A. Toscano, M. Elena García‐Aguilera, Braulio Rodríguez‐Molina

**Affiliations:** ^1^ Instituto De Química Universidad Nacional Autónoma De México, Coyoacán Ciudad de México México

**Keywords:** cocrystals, dynamic crystals, mixed‐solvate cocrystals, responsive crystals, thermosalient effect

## Abstract

Harnessing mechanical motion in molecular crystals is a critical goal for developing novel energy conversion materials. Among known strategies, the ejection of guest molecules from the lattice via the *jumping‐mate* approach offers a direct means of propulsion. Here, we present isostructural cocrystals of indolo[3,2‐*a*]carbazole (ICZ) and (*E*)‐1,2‐di(pyridin‐4‐yl)ethene (BPE) that incorporate solvents such as acetone, ethyl acetate, or tetrahydrofuran. X‐ray diffraction and solid‐state nuclear magnetic resonance (NMR) studies confirmed the presence of solvents or solvent mixtures. Upon heating, these channel‐type cocrystals exhibit a thermosalient effect, with temperature regulation achieved by releasing pure or solvent mixtures. Differential scanning calorimetry and thermogravimetry (DSC–TGA) analyses revealed that the transition temperatures changed progressively, demonstrating controllable thermal actuation through the release of occluded solvents. This work demonstrates a simple yet powerful way to regulate mechanical responses in molecular crystals, thereby advancing the design of responsive energy conversion materials.

## Graphical abstract

Compositionally engineered mixed solvated cocrystals enable modulation of thermosalient actuation. Guest ratio controls thermosalient temperature and propulsion intensity, providing a tunable platform for designing crystalline systems that exhibit stimuli‐responsive mechanical motion.

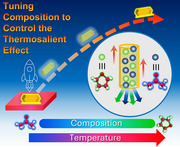

## 1 Introduction

Dynamic crystals are materials that undergo macroscopic mechanical deformation in response to external stimuli, such as light, mechanical stress, or temperature [1, 2]. These crystals attract attention due to their ability to transform heat or light into kinetic energy, known as thermosalience or photosalience, and to exhibit micro or macroscopic motion that can displace them by lengths several times their own size, or deformation, size change, explosions, among other phenomena [3].

Many mechanisms have been reported for jumping crystals, one of which is thermally promoted phase transitions, in which changes in molecular arrangement lead to anisotropic strain accumulation that triggers the jump [4, 5, 6].

Another class of dynamic crystals, less explored, exhibits the fast release of one component from the lattice, known in some reports as “jumping‐mate” [7], and exhibits mechanical motion due to the rapid evacuation of one component, leading to expansion and/or contraction of their dimensions, bending, or destruction. The released molecules, usually in pressurized gas form, generate the thrust to move the crystal.

In the literature, two types of jumping mates have been recognized: the first is the in‐situ formation of the released molecule, most commonly by heat. An earlier example of such a propelled crystal is the reported decomposition of the complex [M(hfac)_2_L_2_] (where M = Cu, Ni, Co, or Mn; hfac = hexafluoroacetylacetonate; and L = nitrosyl nitroxide) [7]. Specifically, the release of nitrosyl nitroxide from the crystal structure generates molecular oxygen, causing the crystal to jump at ambient conditions. Also, crystals of a vinyl azide derivative are reported to decompose under UV light, generating N_2_ [8]. Similar to this behavior, the crystals of the coordination complex (H_3_TIPA)[Fe(C_2_O_4_)_3_]⋅H_2_O, suffer photolysis where the trisoxalate iron(III), [Fe(C_2_O_4_)_3_]^3−^ produces CO_2_, causing mobility upon irradiation [9]. In this area, we recently reported a carbazole–acridine tetrafluoroterphthalic acid cocrystal that, at high temperatures, decarboxylates and releases CO_2_ from its structure, resulting in a thermosalient event [10].

The second jumping mate type is which the released molecule leaves the lattice without reaction, which can be initiated by light or thermal stimuli [11, 12, 13]. Other examples include hydrates [14], organic salts [15], and charge transfer cocrystals [16, 17]. For example, the pyrene tweezer crystals reported by Shibuya et al. showed an interesting jumping behavior when heating, caused by the release of chloroform (CHCl_3_) [18]. In porous structures, the thermal solvent release can also produce a noticeable thermosalient effect, for example, when the hydrogen‐bonded organic framework (HOF) of tetra[2,3]thienylenetetracarboxylic acid evacuates tetrahydrofuran from the pores [19].

It is important to note that, in all the examples mentioned, the loss of a component, and consequently the tuning of the thermosalient effect, cannot be adjusted by changing the composition. To date, the modulation of dynamic behavior in crystals, whether by thermosalience or photosalience, is a frontier in materials science. A seminal work by Barrett et al. shows the control in photosalience through the intensity of the light applied to the cocrystal *cis*–azo(dioxane), which exhibits a progression from subtle photochromism to photomechanical bending, and ultimately to precise laser driven “cold photocarving” enabled by the molecular volatility of dioxane [20].

Additionally, the use of deuterated functional groups or molecules within the lattice can alter the temperature at which the thermosalient effect takes place [21, 22, 23]. Another approach is to prepare solid solutions by doping one material with a second molecule while preserving the initial crystalline structure [24]. This approach has been used to tune luminescence and photomechanical behavior [25], thermal expansion [26, 27], or elastic–plastic behavior [25]. Reported examples of tuning the temperature of thermosalient effect include varying the content of Br or Cl in a coordination zinc complex with bipyridine [28], or through the development of tetrabromobenzene and tetrachlorobenzene solid solutions [29].

The literature indicates that controlling responsiveness in a dynamic crystal upon releasing one or more components is of great interest for future applications. In this context, we introduce a strategy that deliberately incorporates two different solvents within the same crystalline framework to regulate the simultaneous release of the crystal and, consequently, the thermosalient response. To the best of our knowledge, no previous studies have implemented this strategy. Our approach enables systematic tuning of the thermosalience event temperature, providing an additional degree of control over mechanically responsive molecular crystals.

**FIGURE 1:**
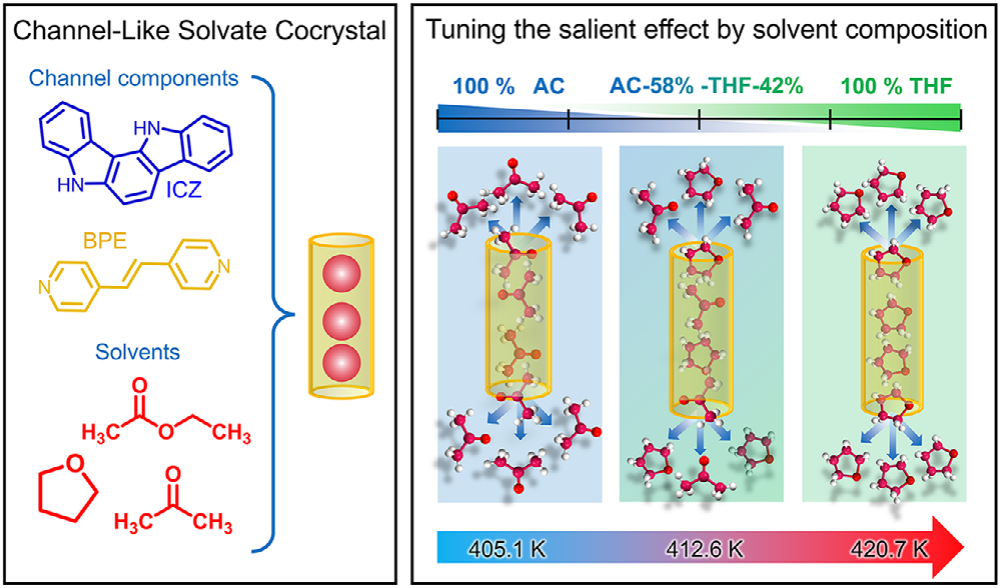
Left: components of the channel‐like solvate cocrystals reported here. Right: representation of the thermosalient effect by solvent mixture composition.

We demonstrate the tuning of the thermosalient effect in three new solvate cocrystals composed of indolo[3,2*‐a*]carbazole (**ICZ**) and (E)‐1,2‐di(pyridin‐4‐yl)ethene (**BPE**), with pure acetone (**AC**), ethyl acetate (**AcOEt**), or tetrahydrofuran (**THF**), or mixtures of them (Figure 1, left). As noted above, the current state of the art for modulating the thermosalient effect (with jumping mates) largely relies on the release of a single guest molecule. Here, we extend this concept beyond simple substitution of the leaving component at various ratios and generate solid solutions that systematically modify the dynamic properties of the pure forms. This compositional approach broadens the horizon of solvent‐assisted thermosalient design and establishes a continuous tuning of the salient temperature.

Variable temperature single‐crystal X‐ray diffraction (VT‐SCXRD), differential scanning calorimetry coupled to thermogravimetry (DSC–TGA), and solid‐state ^2^H NMR experiments enabled us to monitor the behavior of the occluded solvents. Our observation indicates that the crystals form a channeled structure that facilitates solvent release at high temperatures (Figure 1, right). Additionally, we used natural‐abundance or deuterated solvents to achieve detailed control over thermosalient behavior, thereby helping us understand how these jumping crystals work, as described below. The solid solution that varies the content of the “jumping mate” approach enables continuous tuning of the thermosalient temperature, offering a straightforward strategy to control macroscopic crystal motion through compositional design.

## 2 Results and Discussion

### 2.1 Thermosalient Effect on Solvated Cocrystals

**FIGURE 2:**
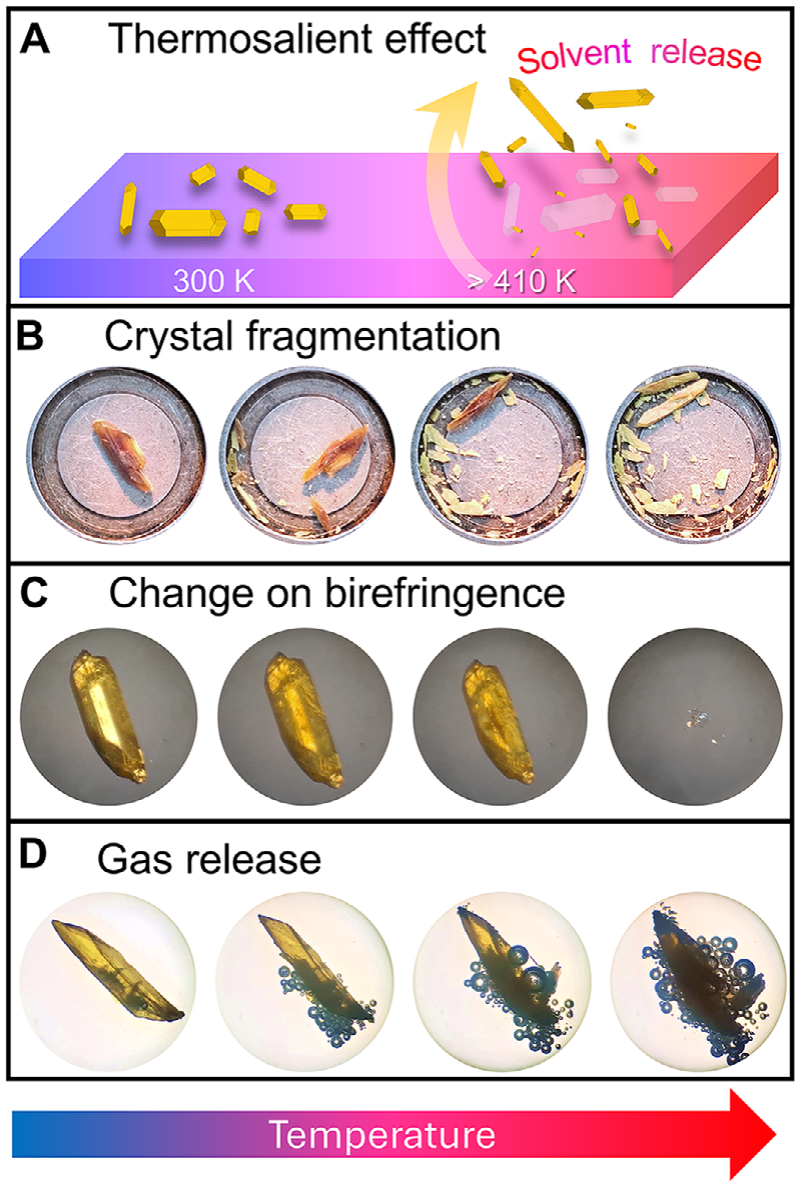
Thermosalient effect of the crystal with **AC**. (A) Schematic representation of thermosalient effect. (B) Crystal fragmentation during heating. (C) Change in birefringence during heating. (D) Crystals covered with oil that evidence the gas release.

The co‐crystallization of **ICZ** and **BPE** in acetone gave well‐defined, prismatic yellow crystals **AC** with variable lengths, from 0.5 mm to 1 cm (See Supporting Information, Cocrystal Synthesis). As part of our standard characterization procedures, we determined the melting point of the crystals using the Fischer–Johns apparatus or a hot‐stage microscope (HSM). When the temperature rose above 130°C (403 K), the crystals jumped and cracked, and these jumps were accompanied by the disintegration of the crystal into smaller particles (Figure 2A, Video S1). The fragments displace violently, in some cases by a few centimeters from the initial position. After the initial thermosalient events, some continued to perform additional jumps and fragmentations, rendering the residual solids opaque. When the temperature exceeded 145°C (418 K), the crystals did not exhibit further thermosalience, and at temperatures between 189–191°C (462–464 K), the crystals finally sublimated and melted.

Additionally, single crystals were analyzed under an HSM equipped with polarized light. At room temperature, the crystal shows uniform birefringence; however, with the temperature rise, at 90°C (363 K), the homogeneity of the birefringence decreases randomly, with dark regions arising and increasing their dimensions and darkness, which was more evident moments before the explosion and jump of the crystal at 132°C (405 K). Detailed observations did not reveal any front face during heating, indicating that the jumps cannot be associated with a martensitic phase transition (Figure 2C, Video S3).

It seemed reasonable to assume that the observed propulsion could be due to the release of at least one component of the lattice. To confirm this hypothesis, we coated the crystals with **AC** with Parabar (cryoprotectant extensively used in crystallography) and heated them over a hot plate. We determined that just below the crystal's fragmentation temperature, small bubbles appeared on the crystal surface at 125°C (398 K). With increasing temperature, the number and volume of bubbles increased, causing them to emerge from internal sites of the crystal and eventually destroying it (Figure 2D).

**FIGURE 3:**
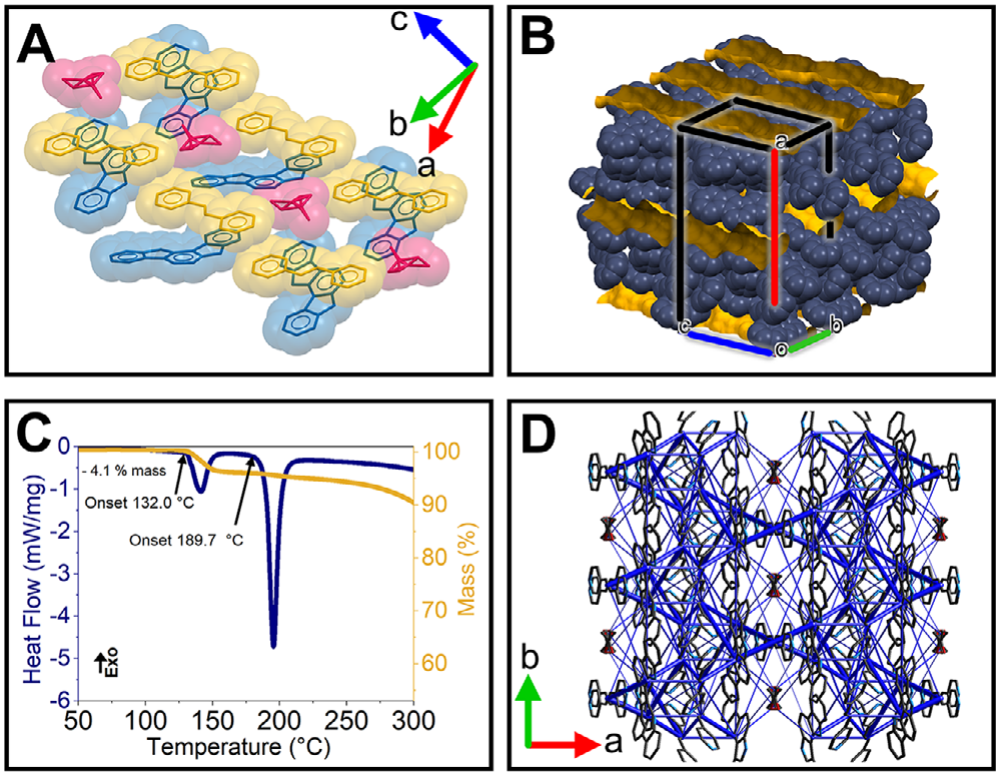
(A) Single crystal structure of the cocrystal with acetone. (B) Inner channel (yellow) resulting from a squeeze refinement. (C) DSC–TGA analyses showing the loss of solvent. (D) Energy framework analysis, highlighting the most representative interactions.

The single‐crystal X‐ray study revealed that the crystal labeled **AC** is a solvate‐cocrystal, consisting of two molecules of indolocarbazole, three molecules of **BPE**, and one molecule of acetone (Table S1), which crystallized in the monoclinic *C*2*/c* space group (Figure 3A). The most relevant aspect of the crystal structure is that the solvent is occluded in channels which are formed by **ICZ** and **BPE** and are stabilized by strong hydrogen bonds between the NH groups of the indolocarbazoles and the N atom of the BPE, along with additional π–π interactions. To estimate and visualize the channel window dimensions and topology, the squeeze tool (Table S9) was applied to the crystal structure [30]. This tool suppresses the solvent molecules during refinement, leaving the crystal with voids, or, in this case, an empty channel. The resulting yellow surface established a wavy channel that extended along the propagation of the crystallographic *c*‐direction (Figure 3B).

In parallel, differential scanning calorimetry and thermogravimetric analyses (DSC–TGA) revealed an endothermic process with an onset at 132°C (405 K), accompanied by a 4.1% mass decrease in a single step. This percentage aligns with the contribution of one equivalent of acetone, which has a theoretical value of 5.2%. We suggest that the slight differences may be due to solvent loss over the elapsed time before the thermal analysis measurement (Figure 3C, Figure S7A).

In a separate experiment to explore if the acetone decomposes now of the jumps, we mounted fresh crystals in a sealed tube, and heated them to a temperature of 150°C. After the thermosalient effect, the collected vapors were analyzed through gas chromatography coupled to mass spectrometry (GC–MS). The corresponding chromatogram (Figure S15, for **AcOEt**, and **THF** the chromatograms are in Figures S16, S17) show that the vapors detected correspond to a simple release of the solvent, without any subproduct.

It is well known that the thermosalient effect is highly anisotropic, which means that the response to heat depends on the face of the crystal that is in contact with the heating (or cooling) surface, and also with crystal dimensions, consistent with other reports on thermosalient crystals (Figure 2B, Video S2) [31, 32].

**FIGURE 4:**
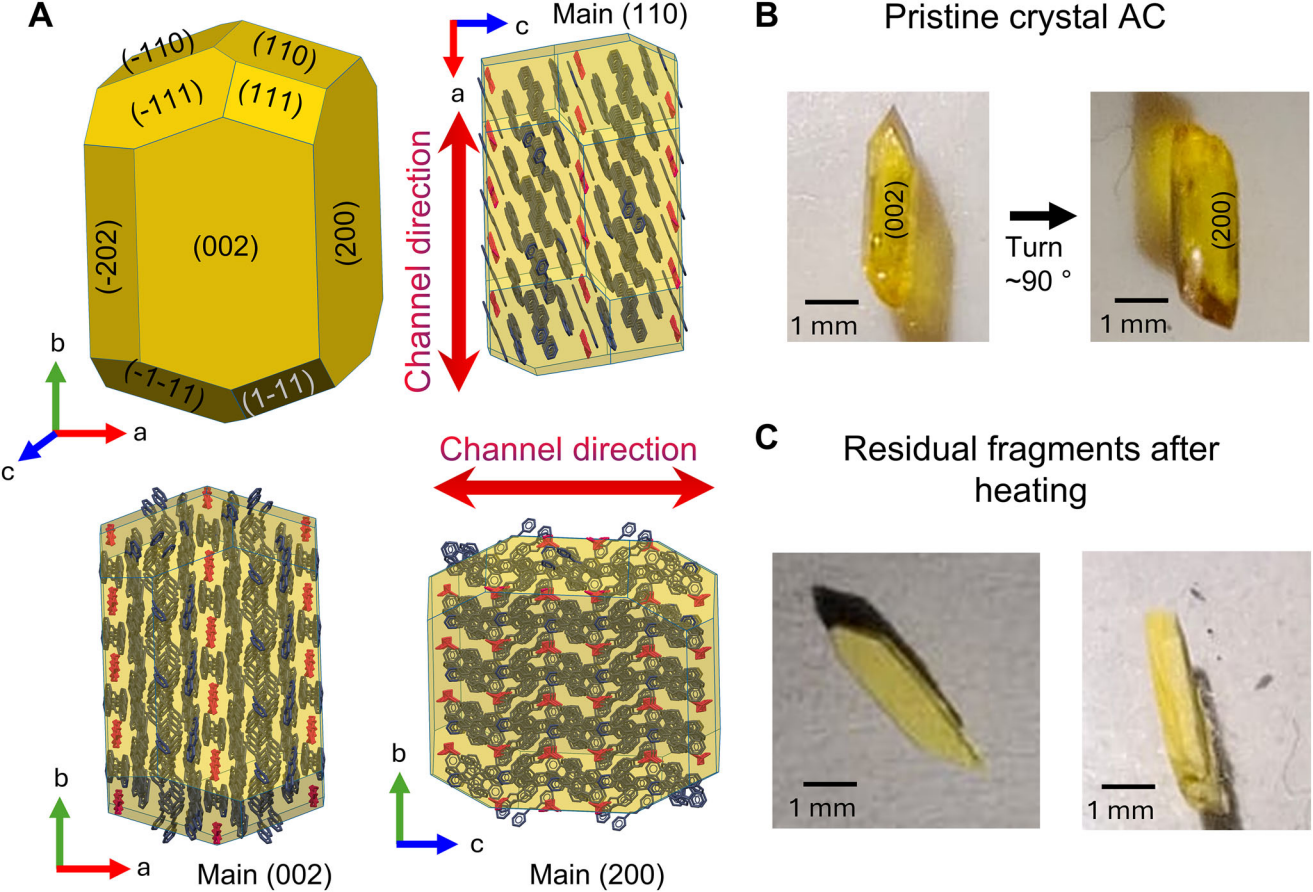
(A) Calculated morphology of the crystal **AC**. (B) Principal faces of the grown crystal. (C) Shape of residual fragments after the thermosalient effect.

To determine the role of the crystal orientation, we employed indexed crystal faces and morphology modeling available in Mercury CCDC (Figure 4) The **AC** crystals have a prismatic habit; during crystallization, they predominantly grow on two faces, the 002 and 200. Figure 4A highlights the habit representation and principal directions: the face (002) has channels perpendicular to the heating surface, whereas the main face (200) is parallel to it. Generally, when the crystal is placed with the channel parallel to the surface, it breaks. Conversely, when it is placed with channels perpendicular to the surface, the crystals move and displace, as illustrated in Figure 4C, and Figure S14A. In both cases, the crystals eventually break, and the fragments can displace, and break 1–3 more times.

Additionally, we explored if the **AC** cocrystal is subject, or not, to a structural transformation accompanied by the loss of acetone (before or after), such transformations can induce thermosalient effect in jumping‐mate systems, [8, 15, 28] or retains their structure as has previously been reported in spironolactone–saccharin hydrate cocrystal [14]. The DSC studies did not show the typical sawtooth profile indicative of a heterogeneous transformation where different domains converted asynchronically [33]. Additionally, the ^13^C CP MAS spectra, recorded between 248 and 323 K, show that the signals of the cocrystal remain stable, which indicates the stability of the cocrystals and the presence of the solvent within the pore. The intensity of the signals for acetone did not vary with the increase in temperature (Figure S11).

Besides, we calculated the energy frameworks based on the crystal structure using CrystalExplorer [34]. The total energy framework follows the shape of the channels; the interactions concentrate in the molecules that form the channels, stabilized by strong π–π interactions and hydrogen bonds, whose principal contributions are dispersive and coulombic, respectively. At the same time, the solvent interacts weakly with the surroundings. According to this analysis, the principal nature of interaction is dispersive, with energy values lower than those of interactions that maintain the channels (Figures 3D and Figures S3A,B).

It is important to note that during heating, crystals undergo thermal expansion (TE), which reflects the distribution and strength of intermolecular interactions within the lattice. We calculated the TE coefficients along the three principal axes in the 100–300 K range using PASCal [35], based on the corresponding unit cell parameters (Table S3). The analysis revealed anisotropic positive expansion (Figure S4A and Table S4), with the largest changes along X3 and X2 (114.4 and 43.9 MK^−^^1^, respectively), and a smaller expansion along the channel direction (21.4 MK^−^^1^) (Figure S4B,C). The TE data complement the energy framework analysis, indicating variations in interaction strength within the lattices.

With all this information, insights suggest that the salient effect is promoted by the rapid release of acetone from the crystal lattice, originating from the nonspecific interaction of the acetone with the channel. As the temperature rises, the internal pressure within the crystal increases, and depending on the face and size of the crystal could exhibit jumps or breaks.

### 2.2 Tuning the Thermosalient Effect Temperature by Changing the Solvent

Once the desolvation of acetone and the weakly nondirectional interactions within the pores were identified as the origin of the macroscopic behavior, it became reasonable to tune the salient temperature by altering the solvent confined within the channels.

**FIGURE 5:**
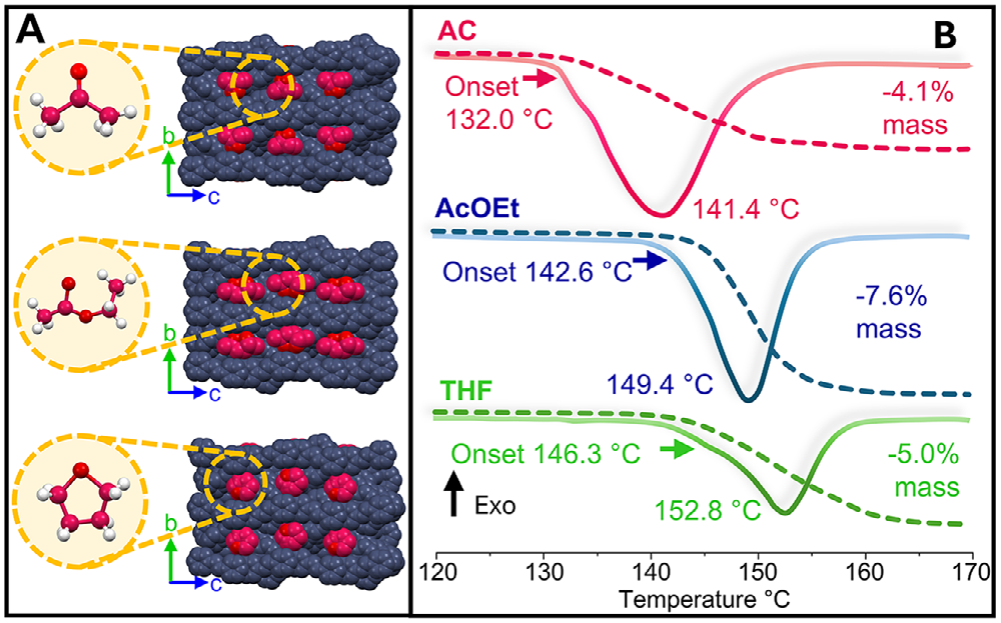
Modulation of the salient effect by solvent occlusion. (A) Single‐crystal X‐ray diffraction of the cocrystals in a space‐filling representation, with the occluded solvent indicated in red; the crystallographic disorder was omitted for clarity. (B) DSC–TGA analyses of the crystals with **AC**, **AcOEt**, and **THF**, showing the gradual increase in the temperature of thermosalience.

This strategy, in principle, would preserve the existence of the channel but could modify the intermolecular interaction of the solvent with its surroundings, thereby provoking a temperature change in the salient effect. To examine this, we cocrystallized (**ICZ**) and (**BPE**) in several organic solvents due to their similar solubility. We were able to grow single cocrystals from ethyl acetate (**AcOEt**), methanol (**MeOH**), acetonitrile (**ACN**), tetrahydrofuran (**THF**), and DMSO/water (**H_2_O**). Under the conditions explored, prismatic yellow crystals with various habits were obtained. Single‐crystal X‐ray diffraction reveals that the solids obtained from **AcOEt, MeOH, ACN, THF,** and **H_2_O** are all solvate cocrystals. Remarkably, only the cocrystals with **AcOEt** and **THF** are isostructural to **AC**, that is, there are channels where the solvent is occluded (Figure 5A; Tables S1 and S5). The remaining solvates **MeOH**, **ACN**, and **H_2_O** do not form a solvent channel, as each exhibits a unique structure (Figure S5A–C and Table S6). All solvated cocrystals were also characterized by powder X‐ray diffraction (Figure S6), DSC–TGA (Figure S7A–F), FTIR ATR (Figure S9), and ss‐NMR ^13^C CP‐MAS (Figure S10). For our purposes, we focused on the salient crystals; however, the structural characterization and description of these solvates are provided in the Supporting Information (Structural description of the solvate‐cocrystal **MeOH**, **H**_**2**_ **O**, and **ACN**).

Only the cocrystals with **EtOAc** and **THF** exhibited jumps and explosions when heated (Figure S14B–D), like those with **AC** (Videos S4–S7). Through these experiments, we confirmed that the thermosalient effect in these solids is a direct consequence of crystal packing; that is, channels are necessary for solvent release in a one‐step process. Our observations also suggest that channel formation is not favored when hydrogen bonds can form between the solvents (**H_2_O**, **ACN**, or **MeOH**) and either conformer, **BPE** or **ICZ**.

Gratifyingly, the presence of tetrahydrofuran or ethyl acetate within the crystal modifies the onset temperature of the thermosalient effect. The DSC–TGA analyses of the cocrystals show that **AC** has the lowest onset at 132.0°C (ca. 405 K), followed by that with **AcOEt** at 144.9°C (ca. 418 K) and with **THF** at 147.7°C (ca. 421 K), indicating also the increased thermal stability of each solvate cocrystal (Figures 5B, S7A–C). The solvent removal shows a good correlation between the experimental and calculated data for the cocrystal with **AcOEt**, at 6.5% (theoretical 7.7%) for the cocrystal with **THF**, and at 5.0% (theoretical 6.4%). After the thermosalient effect, both remaining solids melt over the interval from 189°C to 196°C. Regarding the enthalpy of the endothermic process (area under the endothermic peak), we found that for **AcOEt** is 44.8 kJ mol^−1,^ for **AC** is 32.6 kJ mol^−1^, and for **THF** is 31.6 kJ mol^−1^.

The desolvation temperatures do not scale directly with Δ*H*, suggesting some enthalpy–entropy compensation. Although the **AcOEt** solvate shows the largest enthalpic contribution, it is accompanied by a larger entropy term, leading to a comparable free energy balance at the onset temperature. These values should therefore be regarded as macroscopic descriptors of the desolvation process rather than evidence of a specific microscopic structural mechanism.

With the idea of comparing the frequency of the jumps for each solvate, a *k*‐means clustering approach was carried out, grouping crystals into three size categories (small, medium, and large) according to their length, height, and thickness (A, B, and C). This approach enabled us to perform a size‐dependent comparison of the jump frequency based on the geometric characteristics of the crystals.

*K*‐means clustering revealed natural groupings of crystals primarily governed by the length parameter (A). For crystals grown in **AcOEt**, lengths below 3 mm were classified as small, 3–5 mm as medium, and above 5 mm as large. A similar trend was observed for acetone‐grown crystals, with thresholds of < 3.5 mm (small), 3.5–5 mm (medium), and >5 mm (large). In contrast, crystals grown in **THF** under comparable conditions were systematically smaller, leading to size categories of < 2 mm (small), 2–3 mm (medium), and >3 mm (large).

Due to their size, small crystals have a low probability of jumping, as observed in Figure S13. In contrast, medium‐ and large‐sized crystals are more likely to jump; notably, in the group of large crystals containing AcOEt and acetone, 100% of the crystals jumped. This suggests that once a specific length is exceeded, the jumps will present systematically. For AcOEt, the critical length corresponds to A > 3 mm, and for acetone, A > 4.4 mm

It is essential to note that the temperature at which the salient effects are observed is significantly above the boiling points of **AcOEt**, **AC**, and **THF** (350, 329, and 339 K, respectively), indicating relevant intermolecular interactions between the solvent and the channels. To shed more light on the temperature‐dependent salient effect, we complemented the analysis of energy frameworks for all the cocrystals, focusing on changes in solvent interactions with the channel. In particular, the comparison among solvents agrees with the trend of salient effect: the lowest values of interaction energy coincide with the lowest salient effect temperature, and vice‐versa (Figure S3 and Table S2).

Previous works by Naumov [21], and Angeloski [22], reported that the thermosalient effect in crystals can also be modulated by deuteration of specific molecular fragments. These facts inspired us to explore the use of deuterated solvents, which is an opportunity to fine‐tune the salient effect in crystals. With this in mind, we prepared cocrystals using deuterated solvents, yielding **AC‐*d_6_*** and **THF‐*d_8._***

The DSC–TGA analyses of these samples showed DSC profiles similar to those of their natural abundance analoguess, but with higher desolvation onset temperatures. For the cocrystal with acetone **AC‐*d_6_***, the onset temperature is 407.8 K (134.8°C), an increase of 2.8 K. On the other hand, for the cocrystal with deuterated tetrahydrofuran **THF‐*d_8,_*** the desolvation onset is 421.7K (148.5°C), which is a slight increment of 2.2 K. (Figure S12A,B). The use of deuterated solvent produces measurable shifts in the transition temperature, even when the substituted sites are not directly involved in strong interactions. This behavior is consistent with previous reports and suggests that isotopic substitution modifies the vibrational dynamics of the lattice, increasing the energy required to trigger the structural transformation or desolvation process.

### 2.3 Fine‐Tuning the Salient Effect by the Occlusion of Solvent Mixtures

We then hypothesized that if two different solvent molecules could occupy the channel, the thermosalient effect would change and, consequently, be modulated. Our first approach was to place two solvents, from which the crystals showed different salient behavior: either forming or not forming channels. Within this reasoning, two possible scenarios would occur: (a) the inclusion of both compounds into the channels, and (b) co‐crystallization of only one form due to a solvent competition in the crystallization. We started with a mixture of acetone and methanol (50% v/v) and, after slow evaporation, the resulting crystals showed no salient effect. The melting point of the crystal agreed well with that of the MeOH solvate. After trying different solvent combinations, we concluded that the preferred form for co‐crystallization when using **MeOH**, **H_2_O**, or **ACN** is the non‐salient form due to the creation of strong hydrogen bonds between the solvents and one of the cocrystal components.

The control of the thermosalient effect was achieved by co‐crystallizing **BPE** and **ICZ** with solvent mixtures that favor salient behavior. Given the most noticeable difference in the salient temperature between the cocrystals with **AC** and **THF**, we selected them to prepare three solvent mixtures with nominal v/v ratios of 25/75, 50/50, and 75/25 (**AC**/**THF**). Solution ^1^H NMR of the obtained cocrystals in DMSO‐*d_6_* corroborated the final stoichiometry of the crystal, resulting in 11/89% **AC**/**THF**, 18/82% **AC**/**THF**, and 58/42% **AC**/**THF,** respectively (Supporting Information, Solid solutions, Figure S18 and Table S11).

**FIGURE 6:**
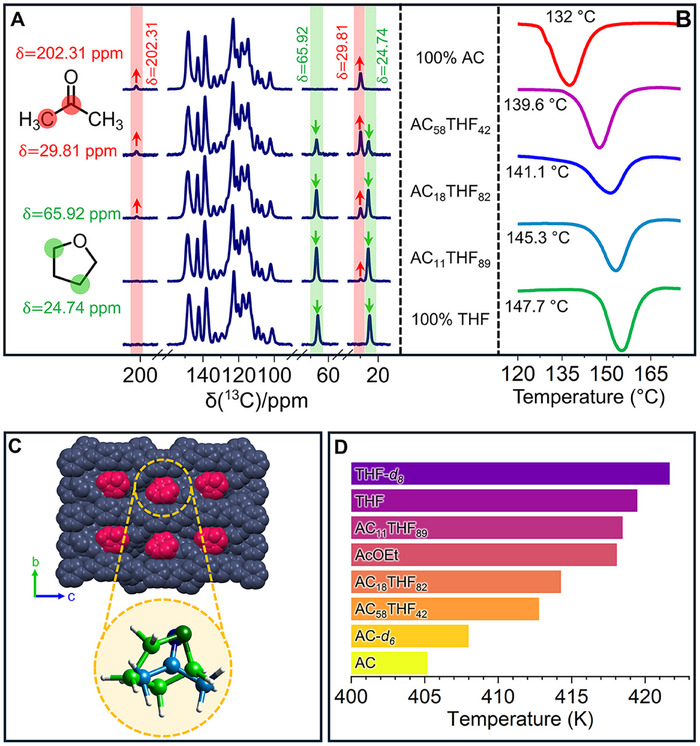
(A) ^13^C CP‐MAS of the corresponding cocrystals with the percentages of the occluded solvents. (B) ^13^C CPMAS of the corresponding cocrystals with the percentages of the occluded solvents. (C) SCXRD of the solid solution AC:THF, in space‐filling representation, with the occluded solvent indicated in red; the crystallographic disorder was omitted for clarity purposes, tetrahydrofuran in green, and acetone in blue. (D) Fine‐tuning the temperature of the salient effect with different compositions of acetone and tetrahydrofuran, either natural abundance or deuterated analogs.

To corroborate the presence of both solvents within the channels, the crystals were analyzed by ^13^C CP‐MAS solid‐state NMR, DSC–TGA, and single crystal X‐ray diffraction (Figure 6A–C). ssNMR can distinguish between different carbons based on their chemical shift and crystallographic environment. First, we obtained the ^13^C spectra of the cocrystals containing 100% **AC** and 100% **THF**. The group of signals in the aromatic region, from 150 to 95 ppm, corresponds to channels composed of **BPE** and **ICZ**. As expected, this region shows notable similarity between the two samples due to crystal iso‐structurality (Figure 6A).

For the cocrystals with pure **AC**, two characteristic signals were identified, one intense signal for the methyl group (─CH_3_) and a second, less intense signal for the carbonyl group (C═O). The ‐CH_3_ appeared at 29.81 ppm, while the >C═O appeared at 202.32 ppm. Conversely, the cocrystal with **THF** showed two signals that correspond to alpha carbon atoms, which appeared at 65.92 ppm, while the beta carbon atoms appeared at 24.74 ppm. In this case, the intensity of each signal is identical to that expected by the same number of attached protons (Figure 6A). Subsequently, the cocrystals with different solvent compositions were analyzed. The intensity of the signals of acetone decreases directly with the reduction of its proportion in the cocrystal. In the same way, the signals corresponding to **THF** grew as its contents increased. The chemical shifts in all explored solid solutions did not show any significant change. Complementarily, the mixed cocrystals were analyzed by DSC–TGA (Figure S8A–C). The results show a mass loss in a simple endothermic process, contrasting with a sample composed of a physical mixture that shows two defined endothermic processes (Figure S8D), and, as anticipated, a shift in the onset temperature of desolvation with a clear trend with solvent content (Figure 6B). Finally, the single‐crystal structure is shown in Figure 6C and Table S8), and, as we expected, it contains both solvents; despite the solvents showing positional crystallographic disorder, we were able to resolve with a 34:66 **AC**:**THF** occupancy.

Figure 6D sumarizes the systematic modulation of the thermosalient temperature across pure, mixed, and deuterated solvates. The transition from **AC** to **THF** in the mixed crystals shows intermediate values that evolve continuously between the two limits. The deuterated analogs (**AC‐*d*_6_**, **THF‐*d*_8_**) follow the same trend but exhibit slightly higher salient temperatures. This behavior demonstrates that precise control over the solvent composition and isotopic identity provides a robust means to tune the thermosalient response in molecular crystals.

### 2.4 Solvent Motion in the Pores

A close inspection of the SCXRD structures of **AC** and **THF** at 298 K revealed that the guest molecules adopt highly disordered positions (Figure S19). In the **AC** and **THF** solvates, two and four disordered guest molecules occupy each cavity, respectively, suggesting that rapid dynamics may occur within the channel.

To unravel the underlying molecular motion, we turned to ^2^H solid‐state NMR (ssNMR) spectroscopy using the deuterated analogs **AC‐*d*_6_** and **THF‐*d*_8_**, a technique suitable for probing ^13^C‐^2^H bond reorientations and their associated rotational trajectory and frequency. Given the pronounced structural disorder, one would intuitively anticipate narrow, isotropic‐like ^2^H signals resulting from unrestricted, dynamically averaged reorientations.

**FIGURE 7:**
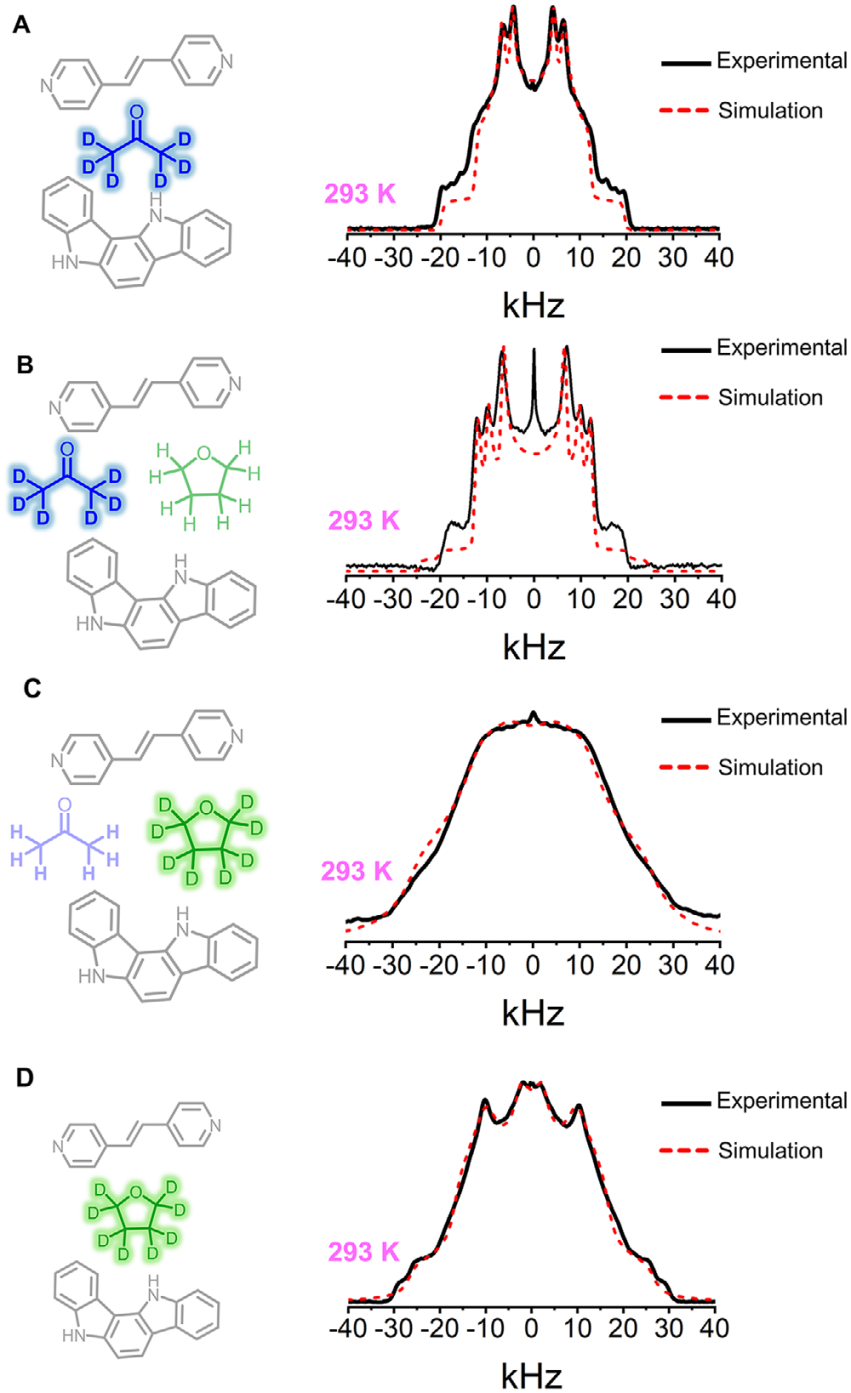
Experimental and simulated ^2^H solid‐state NMR spectra at 293 K for (A) AC‐*d*_6_, (B) AC‐*d*_6_:THF, (C) AC:THF‐*d*_8_, (D) THF‐*d*_8_.

For **AC‐*d*_6_,** the experimental line shape was accurately fitted by considering two fast‐limit motions (FLM > 10^7^ Hz): (i) a 3‐fold reorientation associated with C–D methyl rotation and (ii) an additional 3‐fold reorientation according to the positional disorder of acetone over two sites from the crystallographic data. Variable‐temperature ^2^H ssNMR spectra recorded at 248, 293, and 323 K displayed only subtle changes in the overall line shape (Figure S20A), indicating that the system remains in the fast‐motion regime across this temperature range. All experimental spectra can be described using the same motional model (Figure 7A and Table S15). A superposition of the experimental and fitted spectra reveals slight narrowing of the line shape, consistent with a redistribution of jump rates toward higher frequencies within the fast‐limit regime, reflecting an increase in overall re‐orientational dynamics while preserving the symmetry‐restricted motional modes.

Meanwhile, for the sample with **THF**‐*d*_8_, we fitted the line shape with 4‐fold contributions (Figure 7D and Table S17), which is consistent with the disordered positions observed in SCXRD. The same line shape model was employed at all temperatures, where a slight narrowing was observed at 323 K (Figure S20C).

After studying them individually, we wanted to study solvate cocrystal mixtures with one deuterated solvent. We prepared two new samples, **AC‐*d*_6_:THF** and **AC‐THF‐*d*_8_**, with a nominal concentration of 50%:50%. The resulting deuterium line shape is more complex, particularly for the sample **AC‐*d*_6_‐THF** (Figure 7B and Table S16). This is in line with SCXRD evidence indicating a more disordered structure than that observed with **AC‐*d*_6_** alone, supporting the idea that the occluded molecules interact with one another. Furthermore, a close inspection of these variations (Figure S20B) shows a reduction in signal separation as the temperature increases, indicating greater rotational freedom.

Last, for the sample **AC‐THF‐*d*_8_** same conditions parameters were employed, with a fast (>104 Hz) 4‐fold angular displacements. Decreasing jump angle distributions were considered (Figure 7C and Table S18) an the same narrowing signal trend is observed (Figure S20C).

It is plausible that at high temperatures, the occluded solvent molecules undergo large‐amplitude molecular motions, contributing to the buildup of internal stress that may be released abruptly as a jumping response. Although it does not imply causality, solvent dynamics could play a nonnegligible role in the mechanical response of these crystals.

## 3 Conclusions

Three new channel‐type thermosalient solvate‐cocrystals of indolo[3,2‐*a*]carbazole (**ICZ**) and (*E*)‐1,2‐di(pyridin‐4‐yl) ethene (**BPE**) were obtained using acetone (**AC**), ethyl acetate (**AcOEt**), and tetrahydrofuran (**THF**) as guest molecules. The selected components are key to forming cocrystals with solvent‐filled channels that undergo abrupt mechanical motion upon solvent release at high temperatures. DSC–TGA analyses confirmed that the thermosalience is triggered by the rapid evacuation of solvent molecules, with onset temperatures governed by the strength of dispersive host‐guest interactions.

Mixed‐solvent crystals containing both **AC** and **THF** were successfully obtained and characterized by solid‐state and solution NMR, showing compositional control over the transition temperature. The modulation of onset temperature follows the solvent ratio, producing a single, tunable endothermic event. Overall, these results demonstrate for the first time that the thermosalient behavior of channel‐type cocrystals can be finely tuned by selecting the solvent and adjusting the mixture composition. Our findings also allowed us to propose a mechanism that highlights a new pathway for engineering molecular crystals capable of converting thermal energy into directed mechanical motion with programmable actuation behavior.

## Accession Codes

The following numbers contain the supplementary crystallographic data for this paper: 2497070 (**AC**100 K), 2497072 (**AC**‐298 K), 2497067 (**AcOEt**‐150K), 2497066 (**AcOEt**‐298K), 2497071 (**THF**‐150 K), 2497068 (**THF**‐298 K), 2497073 (**MeOH**), 2497074 (**ACN**), 2497069 (H_2_O), 2504912 (ICZ_BPE_**AC_THF**) These data can be obtained free of charge via www.ccdc.cam.ac.uk/data_ request/cif, or by emailing data_request@ccdc.cam.ac.uk, or by contacting The Cambridge Crystallographic Data Centre, 12 Union Road, Cambridge CB2 1EZ, UK.

## Conflicts of Interest

The authors declare no conflicts of interest.

## Supporting information




**Supporting File 1**: Synthetic details, NMR characterization, thermal stability analyses, powder X‐ray diffraction studies, crystallographic information and analyses, and solid‐state NMR studies.

**Supporting File 2**: anie71541‐sup‐0002‐Data.zip.

**Supporting File 3**: anie71541‐sup‐0003‐VideoS1.mp4.

**Supporting File 4**: anie71541‐sup‐0004‐VideoS2.mp4.

**Supporting File 5**: anie71541‐sup‐0005‐VideoS3.mp4.

**Supporting File 6**: anie71541‐sup‐0006‐VideoS4.mp4.

**Supporting File 7**: anie71541‐sup‐0007‐VideoS5.mp4.

**Supporting File 8**: anie71541‐sup‐0008‐VideoS6.mp4.

**Supporting File 9**: anie71541‐sup‐0009‐VideoS7.mp4.

## Data Availability

The data that support the findings of this study are available in the supplementary material of this article.
