# Supplementary material for: Crystals as Rockets: Modulation of the Salient Temperature in Cocrystals by Solvent Mixture Composition
Source: Angew Chem Int Ed Engl. 2026 Mar 18;65(18):e26010. doi: 10.1002/anie.202526010 (PMC13110762; doi:10.1002/anie.202526010)
Supplement: Supplementary file 1 — Supporting File 1: Synthetic details, NMR characterization, thermal stability analyses, powder X‐ray diffraction studies, crystallographic information and analyses, and solid‐state NMR studies. [file ANIE-65-e26010-s005.pdf]

# *Supporting information*

# Crystals as rockets: modulation of the salient temperature in cocrystals by solvent mixture composition

Ernesto A. Hernández-Morales, [a] Dayra Barreto-Hernández, [a] Dazaet Galicia-Badillo,[a] Rubén A. Toscano, [a] M. Elena García-Aguilera[a]*, Braulio Rodríguez-Molina[a]*.

[a] Ernesto A. Hernández-Morales, Dayra Barreto-Hernández, Dazaet Galicia-Badillo, Rubén A. Toscano, M. Elena García-Aguilera, Braulio Rodríguez-Molina.
Instituto de Química, Universidad Nacional Autónoma de México, Ciudad de México 04510, México
E-mail: mgarciaa@iquimica.unam.mx, brodriguez@quimica.unam.mx

## Materials and methods

The commercial reagents 1,3-cyclohexanedione, phenylhydrazine hydrochloride, and solvents AcOEt, acetone, THF, MeOH, and ACN are of reactive grade and were purchased from Sigma Aldrich or Teqsiquim. Parabar® oil was purchased from HAMPTON RESEARCH.

*Parabar® Specifications*

Parabar® is a cryoprotectant for both small and large molecules employed extensively in crystallography. The density of the material is 0.88 kg/dm$^3$. The composition of Parabar is a blend of polyisobutylenes with a Staudinger molecular weight of about 20,000, containing about 20% polymer. Refractive index 1.48487 at 20°C. Kinematic viscosity 641 cSt at 100°C. Kinematic viscosity ratio 2.05 at 100°C. Synonyms used in the crystallographic literature: Paratone® N, Paratone® 8277, Infineum V8512.

Solution $^{1}H$ and $^{13}C$ NMR spectra were obtained with a Bruker AVANCE III HD 700 MHz at room temperature. Spectroscopic data is referenced to acetone-$d_6$ ($^{1}H$: δ = 2.609 ppm, quint. $^{13}C$: δ = 206.26 ppm). Solid-state $^{13}C$ CP-MAS spectra were obtained using a Bruker Avance III 500 MHz spectrometer at 11.7 T field (500 MHz 1H Larmor frequency) equipped with a PH MAS DVT 500S1 BL3.2 N-P/F-H probe. Approximately 50 mg of fresh crystals were analyzed (d1 = 15 s, ns = 2160, p15 = 2.5 ms, AT = 27 ms, SP = 20 kHz, T = 20 °C, 80 kHz decoupling field). FTIR spectra were measured in a Thermo Scientific equipped with a diamond tip in the spectral window from 4000 to 400 $cm^{-1}$. High-Resolution Mass Spectrometry was obtained on a JEOL JMS-AccuTOF JMS-T100LC spectrometer using Direct Analysis in Real Time (DART) ionization. DSC and TGA were obtained simultaneously by a Netzsch STA 449 F3 Jupiter under $N_2$ atmosphere with a heating ramp of 20 °C/min. Samples were put in an aluminum crucible with a hole in the cap. Powder X-Ray diffraction (PXRD) data were collected with a Bruker D8 Advance diffractometer, using CuK (1.54183 Å) radiation source and Linxeye detector. It was used with a Bragg-Brentano configuration in the interval 2θ: 5-50° with a step size of 0.03°. Crystal structure was obtained through single crystal X-Ray Diffraction in a Bruker D8 Venture diffractometer, micro-focus X-ray with a MoKα (0.71073 Å) radiation source. All non-hydrogen atoms were refined anisotropically. Hydrogen atoms were placed in calculated positions and refined using a riding model. Thermosalient effect was tested under a hot stage LIKMAN 420 System, with Olympus BX43 microscope, the photos and videos were recorded employing a digital camera Samsung Galaxy A55, the video was recorded at 60 fps.

**Complementary videos**

- Video S1: **AC** cocrystals heating on a hot plate covered with glass.
- Video S2: **AC** cocrystal heating under polarized light.
- Video S3: **AC** cocrystal covered with oil and heating.
- Video S4: **AC** cocrystals displacements and jumps.
- Video S5: **AcOEt** cocrystals displacements and jumps.
- Video S6: **AcOEt** final jump.
- Video S7.**THF** cocrystal during heating.

**Synthesis of 5H,12H-indolo[3,2-*a*]carbazole (ICZ)**

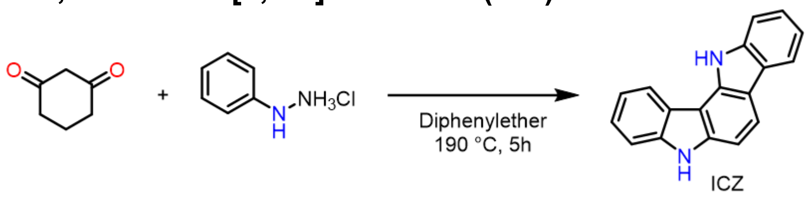


**Scheme S1.** Synthesis of ICZ.

**ICZ** was synthesized following the method reported in **Reference S1**[1] with minor modifications. In a pressure vessel, 1,3-cyclohexanedione (1.00 g, 8.92 mmol, 1.0 eq) and phenylhydrazine hydrochloride (2.97 g, 20.51 mmol, 2.3 eq) were dissolved in 15 mL of diphenyl ether. The mixture was heated at 190 °C for 5 hours. Then, the reaction was completed and cooled at room temperature, and 100 mL of ethyl acetate was added to the mixture and filtered. The liquid was concentrated in vacuum purified twice through column chromatography (first column eluent Hex: AcOEt 90/10 and second column eluent DCM) to obtain a white solid (400 mg, 17.5%). $^{1}$H NMR (700 MHz, Acetone-$d_6$) δ 10.91 (s, 1H), 10.60 (s, 1H), 8.55 (d, $J$ = 7.8 Hz, 1H), 8.16 (d, $J$ = 8.4 Hz, 1H), 8.13 (d, $J$ = 7.7 Hz, 1H), 7.62 (dd, $J$ = 16.4, 8.0 Hz, 2H), 7.42 (t, $J$ = 8.9 Hz, 2H), 7.35 (t, $J$ = 7.1 Hz, 1H), 7.29 (t, $J$ = 7.4 Hz, 1H), 7.23 (t, $J$ = 7.4 Hz, 1H). $^{13}$C NMR (176 MHz, Acetone-$d_6$) δ 140.56, 140.47, 140.06, 135.17, 125.16, 125.09, 124.36, 122.81, 121.67, 120.07, 119.85, 119.64, 119.15, 116.23, 111.79, 111.59, 107.95, 104.5.

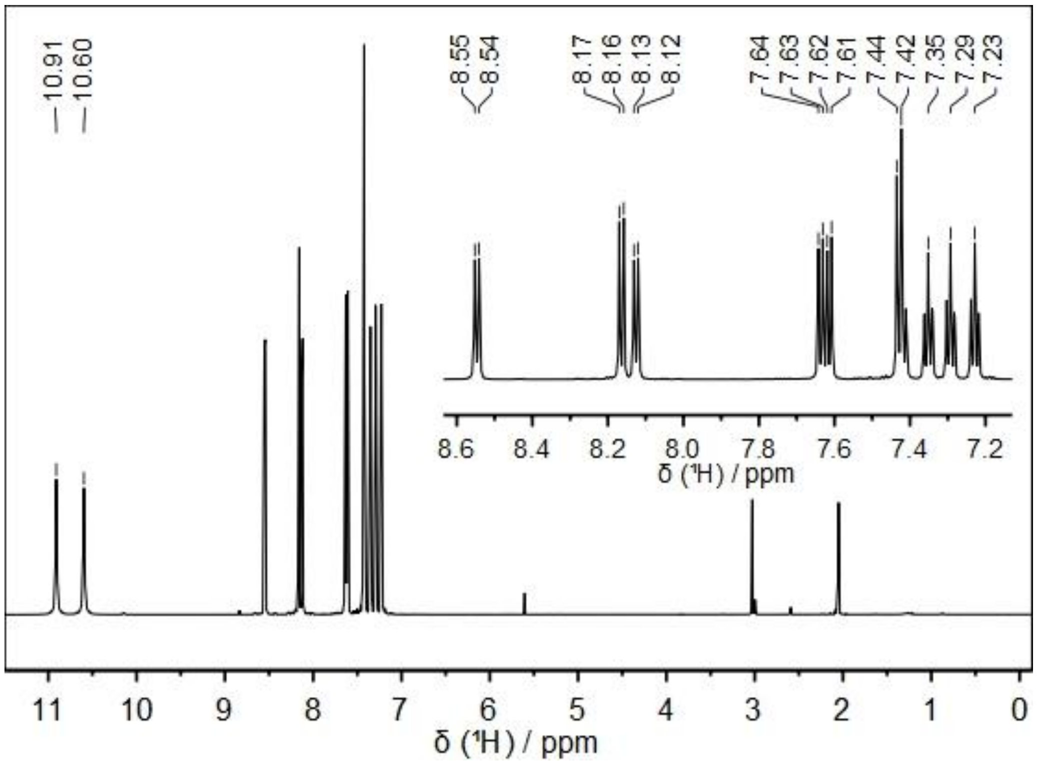


**Figure S1.** $^{1}$H NMR of indolo[3,2-*a*]carbazole (700 MHz; acetone-$d_6$; 298.3 K).

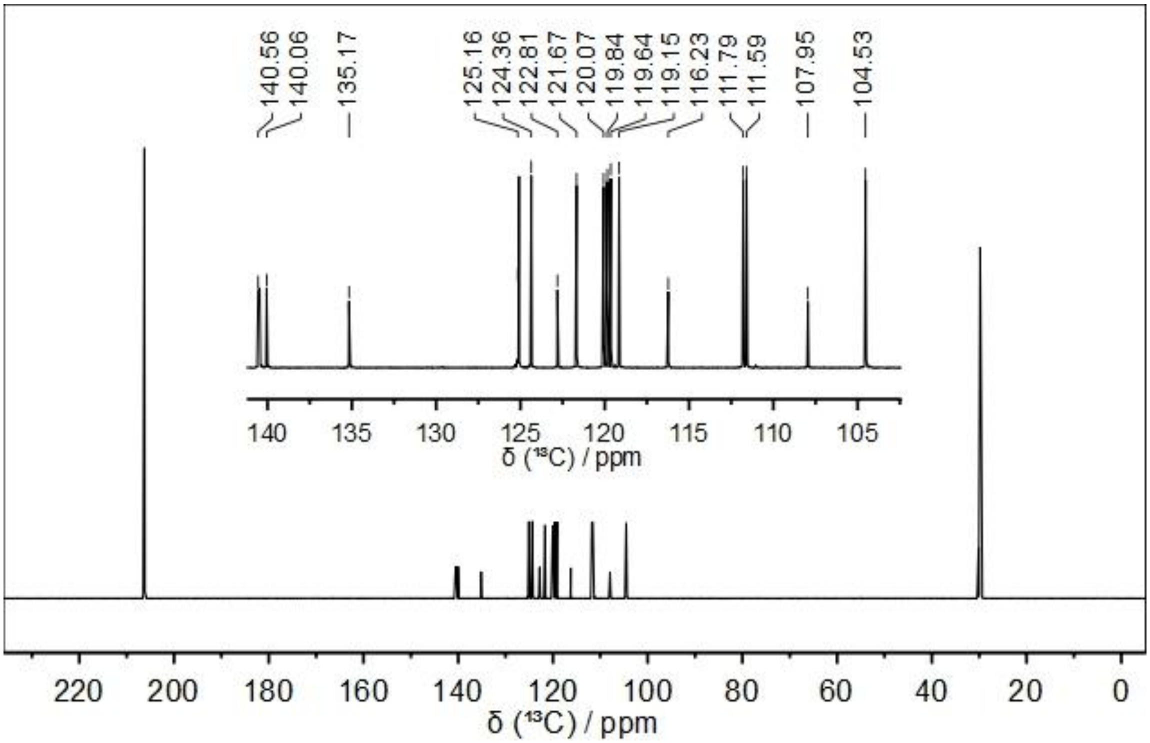


**Figure S2.** $^{13}$C NMR of indolo[3,2-*a*]carbazole (176 MHz; acetone-$d_6$; 298.3 K).

## Cocrystal Formation

Initial experiments for cocrystallization were made as follows: in a 4mL vial, 10 mg of **ICZ** (1 eq, 0.04 mmol) and 7 mg of **BPE** (0.04 mmol, 1 eq), afterward, 2 mL of selected solvent (**AcOEt**, **AC**, **MeOH**, **DMSO** or **ACN**), was added, then the vial was sealed and heated to 85°C until complete solubilization. The cap was slightly open and left to cool and evaporate slowly. After 1 or 2 days, yellow crystals were recovered from the vial and analyzed by single-crystal X-ray Diffraction. In addition to this analysis, the correct stoichiometry is presented and summarized in **Table S1**.

**Table S1*.*** Stoichiometry of the ICZ: **BPE** Solvate Cocrystal and Composition.

| Cocrystal-solvate | ICZ (eq) | ICZ (%) | BPE (eq) | BPE (%) | Solvent (eq) | Solvent (%) |
|---|---|---|---|---|---|---|
| **AcOEt** | 2 | 44.68 | 3 | 47.64 | 1 | 7.68 |
| **Acetone** | 2 | 45.87 | 3 | 48.93 | 1 | 5.20 |
| **THF** | 2 | 45.31 | 3 | 48.32 | 1 | 6.37 |
| **MeOH** | 4 | 43.35 | 7 | 53.94 | 2 | 2.71 |
| **ACN** | 4 | 43.03 | 7 | 53.53 | 2 | 3.44 |
| **$H_2O$** | 4 | 44.21 | 7 | 55.01 | 1 | 0.78 |

## Optimized procedure for cocrystallization for Chanel-type Solvated cocrystals (AC, AcOEt, and THF)

In a 50 mL beaker, 50 mg of **ICZ** (0.20 mmol, 2 eq), 55 mg of **BPE** (0.30 mmol, 3 eq), and 5 mL of the corresponding solvent were added. The beaker was covered with a perforated aluminum foil and heated to 85 °C until complete solubilization of solids. The beaker was left to cool to room temperature and evaporate the solvent, and prismatic yellow crystals were obtained.

- **AcOEt:** Average evaporation time: 6 hours, recovered: 76 mg of crystals (68% yield).
- **AC:** Average evaporation time: 4 hours, recovered 93 mg of crystals (85 % yield).
- **THF:** Average evaporation time: 6 hours, recovered 79 mg of crystals (72% yield).

**Synthesis of hydrated-cocrystal $H_2O$**

In a 4 mL vial, 40 mg of **ICZ** (0.16 eq, 2 eq) and 44 mg of **BPE** (0.28 mmol, 3 eq), and 1 mL of DMSO were added. The vial was sealed and heated to 85 °C until complete solubilization of solids. Afterwards, the vial was opened and left to crystallize for 2-3 weeks; prismatic yellow crystals were obtained.

- **$H_2O$:** Recovered 78 mg of crystals (86% yield).

**Energy frameworks analysis**

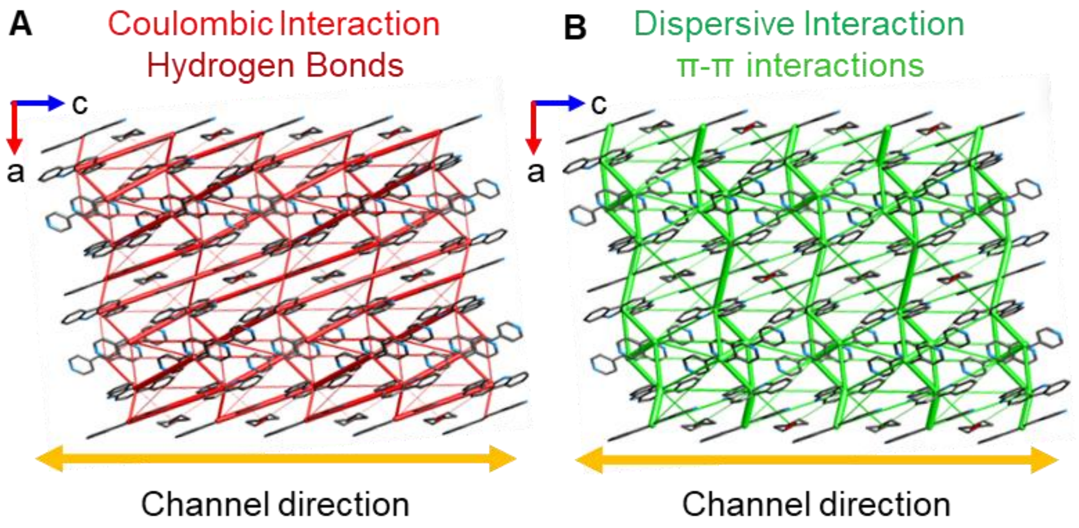


**Figure S3**. Energy frameworks of **AC**, A) coulombic interactions, B) dispersive interactions.

**Table S2.** Summary of energy framework analysis for channel-like solvate cocrystals. Principal interactions, aromatic (π-π), hydrogen bond (HB), and their energy values.

| | | Energy ( kJ $mol^{-1}$) | | |
|---|---|---|---|---|
| Interaction | NATURE | AC | AcOEt | THF |
| π π | Dispersive | -51.8 | -48.8 | -49-9 |
| HB | Coulombic | -34.8 | -33.9 | -34.7 |
| Solvent | Dispersive | -8.5 | -11.9 | -12.1 |

## Thermal expansion (TE) of AC

For cell parameters: X-ray diffraction data were collected in a Bruker D8 Advance diffractometer using MoKa radiation (0.71073 Å), 50 kV, 1 mA, 360 frames for a total of 0.1 h. Unit cell parameters are based upon the refinement of the XYZ-centroids of reflections at variable temperature with uncertainty σ =2 K.

**Table S3.** Unit cell parameters at different temperatures of the cocrystal with acetone.

| Temperature (K) | a (Å) | b (Å) | c (Å) | α (°) | β (°) | γ (°) | Volumen (Å³) | Reflections | 2θ (°) |
|---|---|---|---|---|---|---|---|---|---|
| 101 | 30.433(3) | 10.2580(7) | 18.7044(17) | 90 | 98.471(3) | 90 | 5775.4(14) | 4971 | 2.733-46.82 |
| 150 | 30.523(2) | 10.2810(6) | 18.7125(16) | 90 | 98.575(3) | 90 | 5806.6(12) | 4745 | 2.733-45.52 |
| 175 | 30.595(3) | 10.2913(7) | 18.7229(17) | 90 | 98.630(3) | 90 | 5828.4(13) | 4590 | 2.711-44.38 |
| 200 | 30.671(3) | 10.2997(8) | 18.734(2) | 90 | 98.702(4) | 90 | 5850.0(17) | 4342 | 2.729-42.61 |
| 225 | 30.795(3) | 10.3189(9) | 18.763(2) | 90 | 98.760(4) | 90 | 5892.9(18) | 4120 | 2.723-42.40 |
| 250 | 30.880(5) | 10.3244(12) | 18.769(3) | 90 | 98.838(5) | 90 | 5913.(2) | 3807 | 2.714-42.30 |
| 275 | 30.998(7) | 10.3370(19) | 18.792(4) | 90 | 98.891(6) | 90 | 5949.(4) | 3606 | 2.704-40.48 |
| 298 | 31.103(10) | 10.344(3) | 18.802(6) | 90 | 98.942(7) | 90 | 5976.(5) | 3206 | 2.682-40.12 |

**Table S4**. Linear TE coefficients of **AC** in the range of 100 to 298 K, where σ denotes the error.

| **Axes** | **α ($MK^{-1}$)** | **σα ($MK^{-1}$)** | **Orthogonal plane to the expansion direction** |
|---|---|---|---|
| **X1** | **21.4** | **2.9** | **(2 0 9)** |
| **X2** | **43.9** | **1.1** | **(0 1 0)** |
| **X3** | **114.4** | **6.4** | **(8 0 -3)** |
| **V** | **182.4** | **9.1** | **NA** |

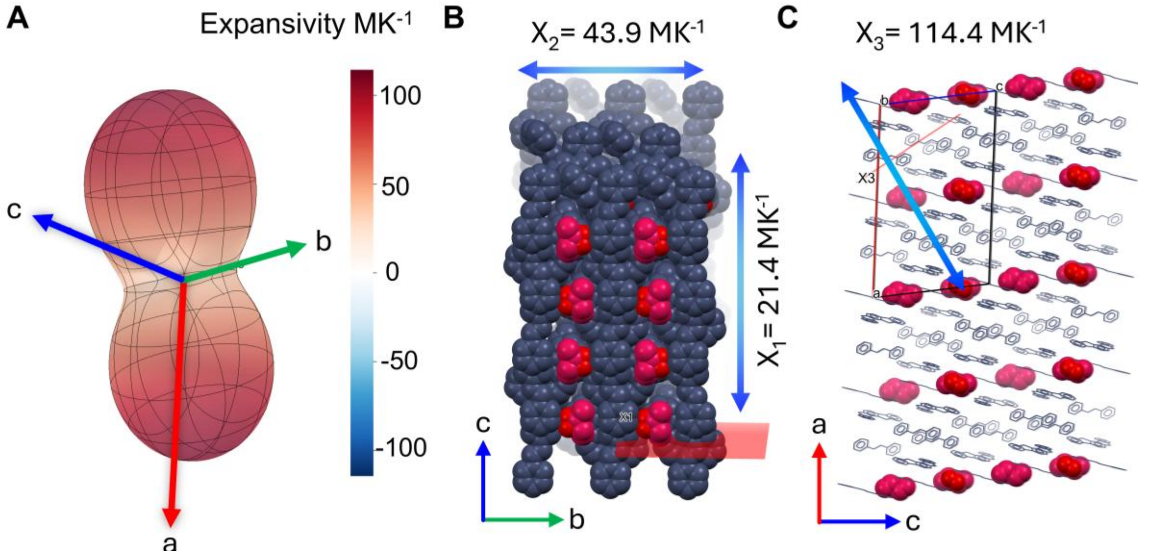


**Figure S4.** Thermal expansion of **AC**, A) Indicatrix, B) $X_1$ and $X_2$ axes directions, C) $X_3$ axis direction**.**

## Single Crystal X-ray diffraction of MeOH, ACN, and $H_2O$

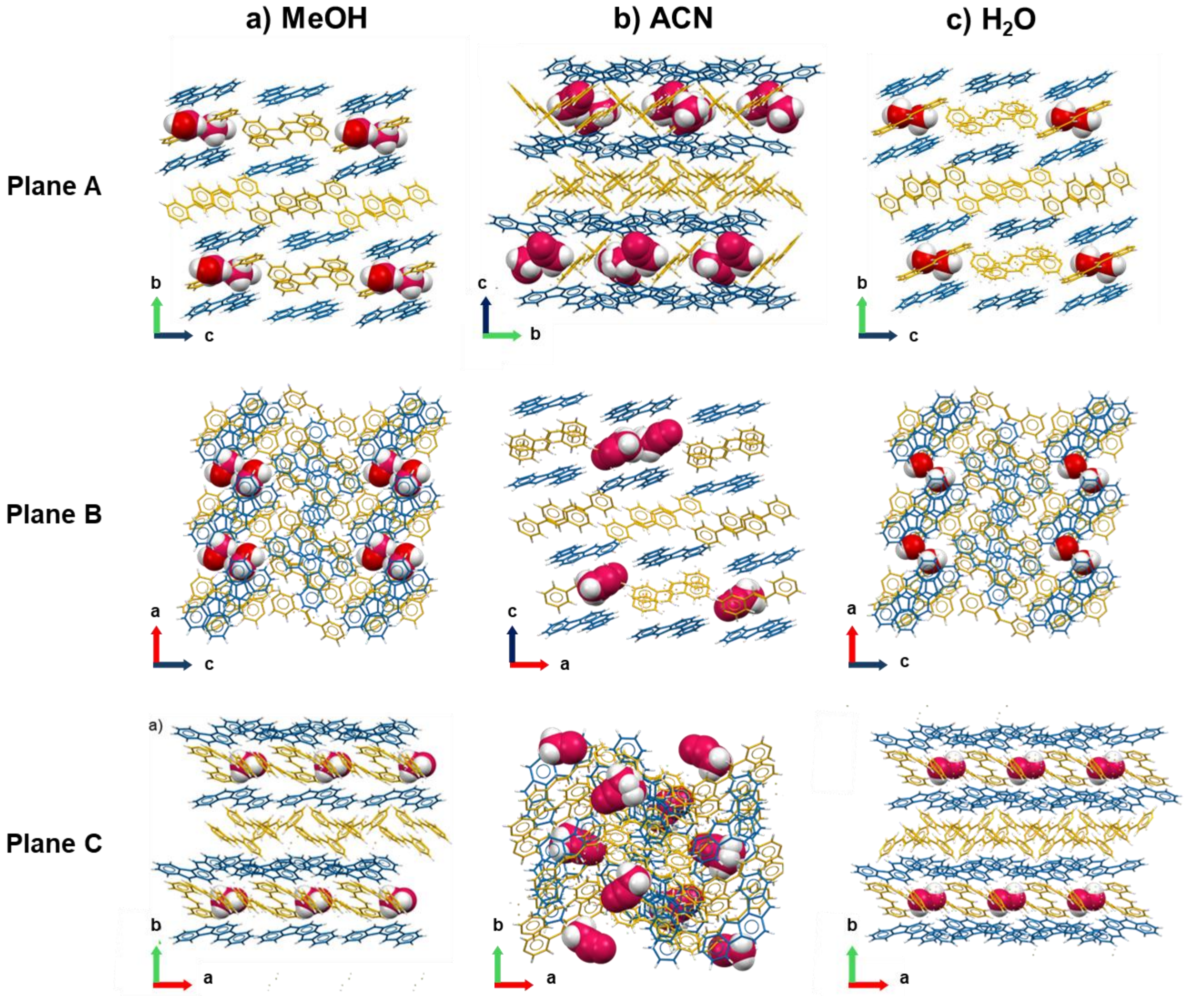


**Figure S5.** Single crystal structures along different views of the planes for a) **MeOH**, b) **ACN**, and c) **$H_2O$**.

**Structural description of the Solvate-cocrystals MeOH, $H_2O$, and ACN**

Cocrystal with **MeOH** crystallizes in the triclinic P-1 space group; the asymmetric unit (Z') consists of two molecules of **ICZ**, 3.5 molecules of **BPE**, and one molecule of methanol. Indolocarbazole forms two strong hydrogen bonds with **BPE** which forms an alternated chain with 1.99(3) Å and 1.96(2) Å of length and angles 174(3)° and 171(2)° respectively, a third of the molecules of **BPE** form a hydrogen bond with **MeOH** which interrupts the chain, also **MeOH** forms a dimer with another molecule of **MeOH** interacting by a non-classical hydrogen bond of 1.514 Å of length and angle 168.2°. Moreover, aromatic interactions are present between **ICZ** and **BPE** CH-$\pi$, $\pi$–$\pi$, and $\pi$–$\pi$ among **BPE**: 3.638(3) Å and 3.959(3) Å of length and angle of 70.3(1)° and 57.2(1)°, respectively. On the other hand, the cocrystal with **ACN** crystallizes in the triclinic $P2_1/n$ space group; two molecules of **ICZ**, 3.5 molecules of **BPE**, and one **ACN** conform the asymmetric unit (Z'). Indolocarbazole interacts with **BPE** through hydrogen bonds: 1.99(2) Å and 2.07(1) Å of length and angles 170(1)° and 164(1)° respectively, but not all pyridine moieties form hydrogen bonds, instead, some of them interact by CH-$\pi$ interactions with indolocarbazole, furthermore, the **BPE** trends to for dimers by $\pi$–$\pi$ stacking between another molecule of **BPE**. **ACN** interacts with at least 3 **ICZ** molecules and two **BPE** molecules by weak CH-$\pi$ interactions.

Instead of **DMSO** being the selected solvent, this solvent was not anhydrous, and it contained water **$H_2O$**, which explains that **BPE** and **ICZ** cocrystallize as a hydrate. The crystal has the triclinic P-1 space group, 3.5 molecules of **BPE**, 2 molecules of **ICZ**, and one molecule of water conform Z'. The **ICZ** and **BPE** interact through three strong hydrogen bonds with distances NH-N 1.85(3) Å, 1.94(3) Å, 1.97(4) Å, and 2.07(3) Å and angles of 171(3)°, 166(3)°, 173(3)°, 158(3)°, respectively. Also, aromatic interactions are observed between **BPE** and **ICZ** by CH-$\pi$ and between two molecules of **BPE** by $\pi$–$\pi$ 3.573 Å and 73.93°. Water molecules interact with **BPE** by a strong hydrogen bond with a distance NH-N of 2.15(2) Å and an angle of 154(2)°. And no channel form was observed in the structure.

**Powder X-ray Diffraction**

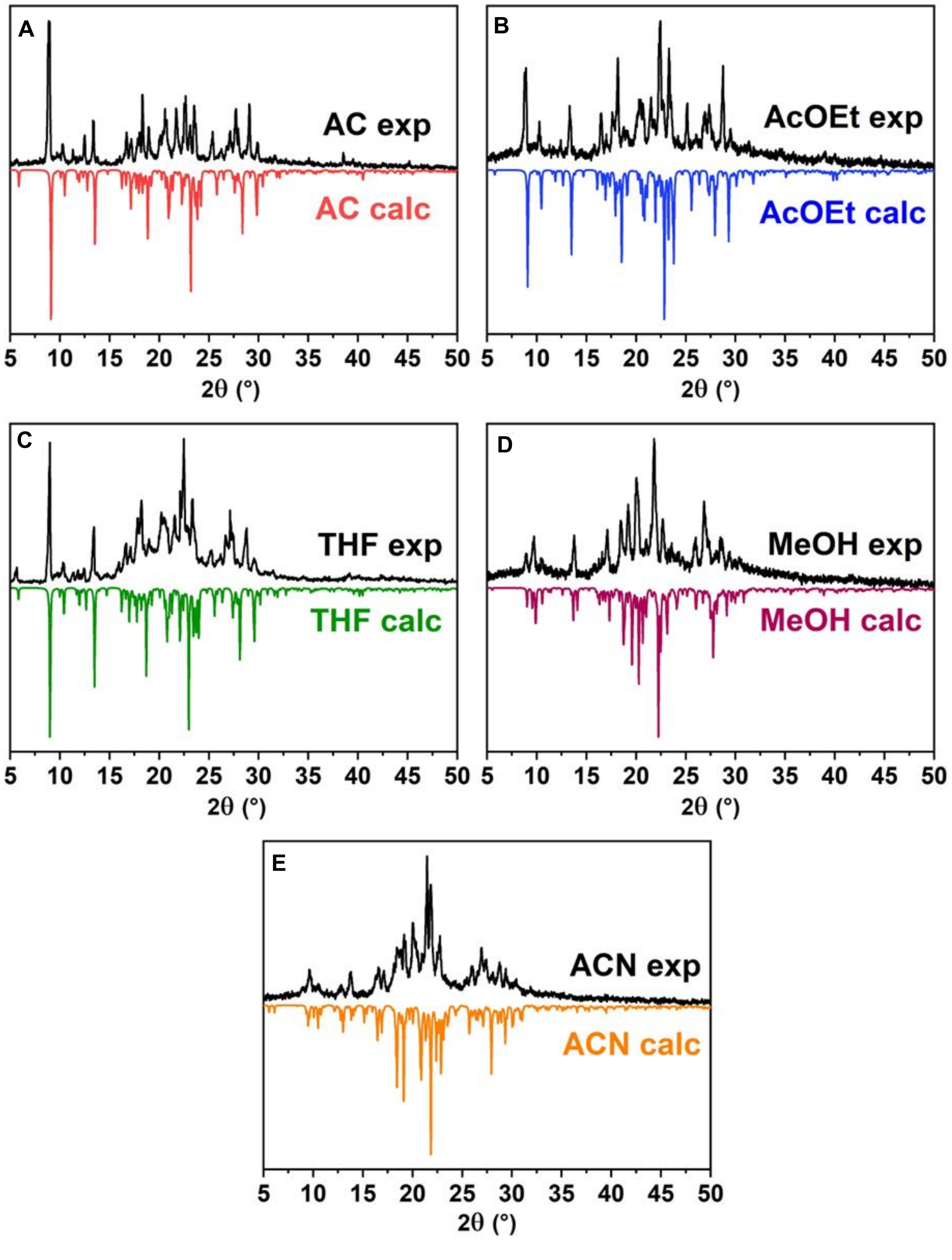


**Figure S6.** Comparison of experimental (black line) and calculated (color line) PXRD of cocrystal with A) **AC**, B) **AcOEt**, C) **THF**, D) **MeOH**, and E) **ACN**.

## DSC-TGA of pure solvated cocrystals

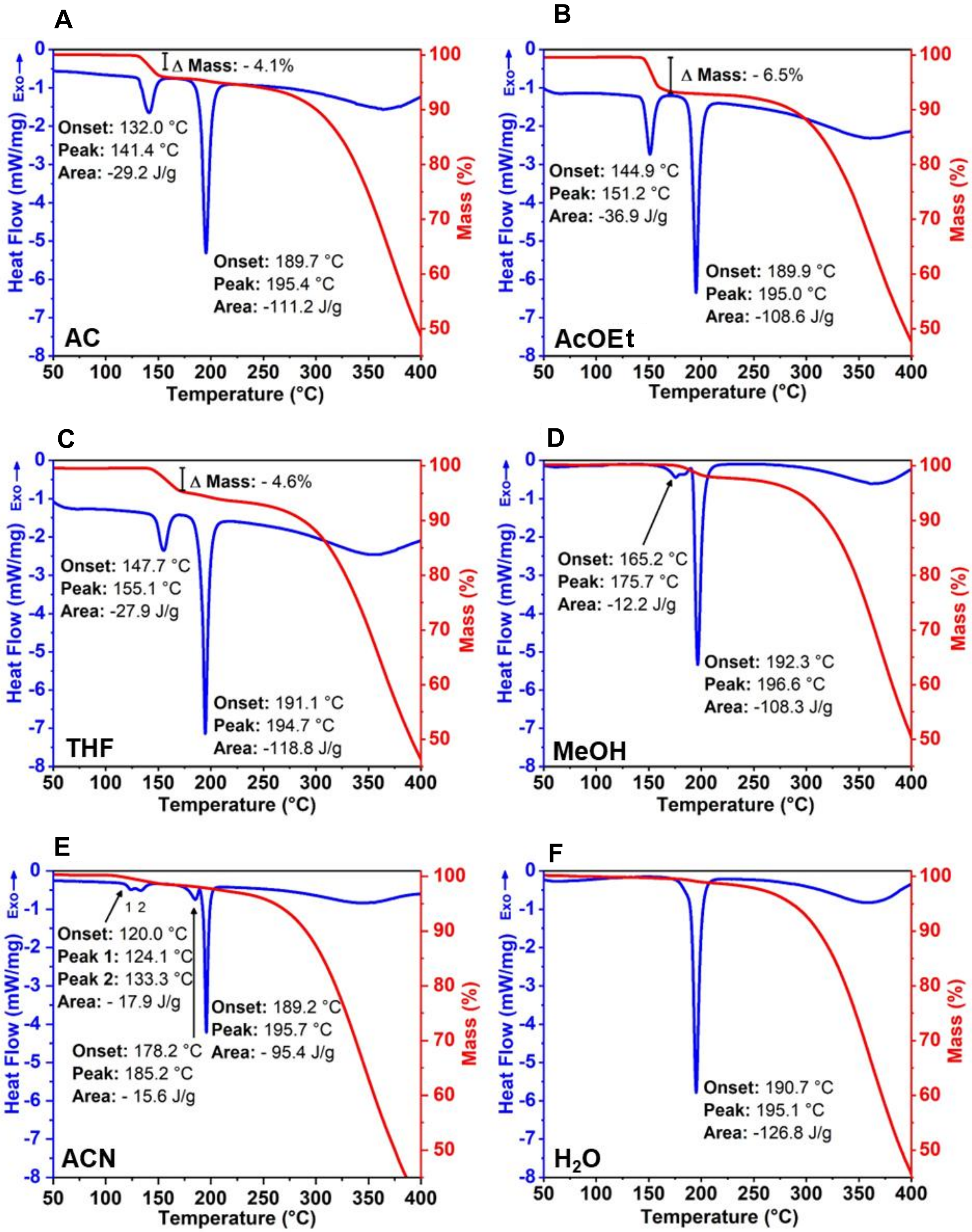


**Figure S7.** DSC (blue line) and TGA (red line) analysis of solvate cocrystals (Single crystals) with A) **AC** B) **AcOEt**, C) **THF**, D) **MeOH**, E) **ACN**, and F) **$H_2O$**.

**Table S5**. Enthalpy and entropy involved in the thermosalient effect.

| | ΔH (kJ/mol) | $T_{desolvation}$ (K) | ΔS (J/mol K) |
|---|---|---|---|
| **I-AcOEt** | 44.8 | 418.05 | 107.0 |
| **I-Ac** | 32.6 | 405.15 | 80.5 |
| **I-THF** | 31.6 | 240.15 | 75.0 |

## DSC-TGA of mixed solvated cocrystals

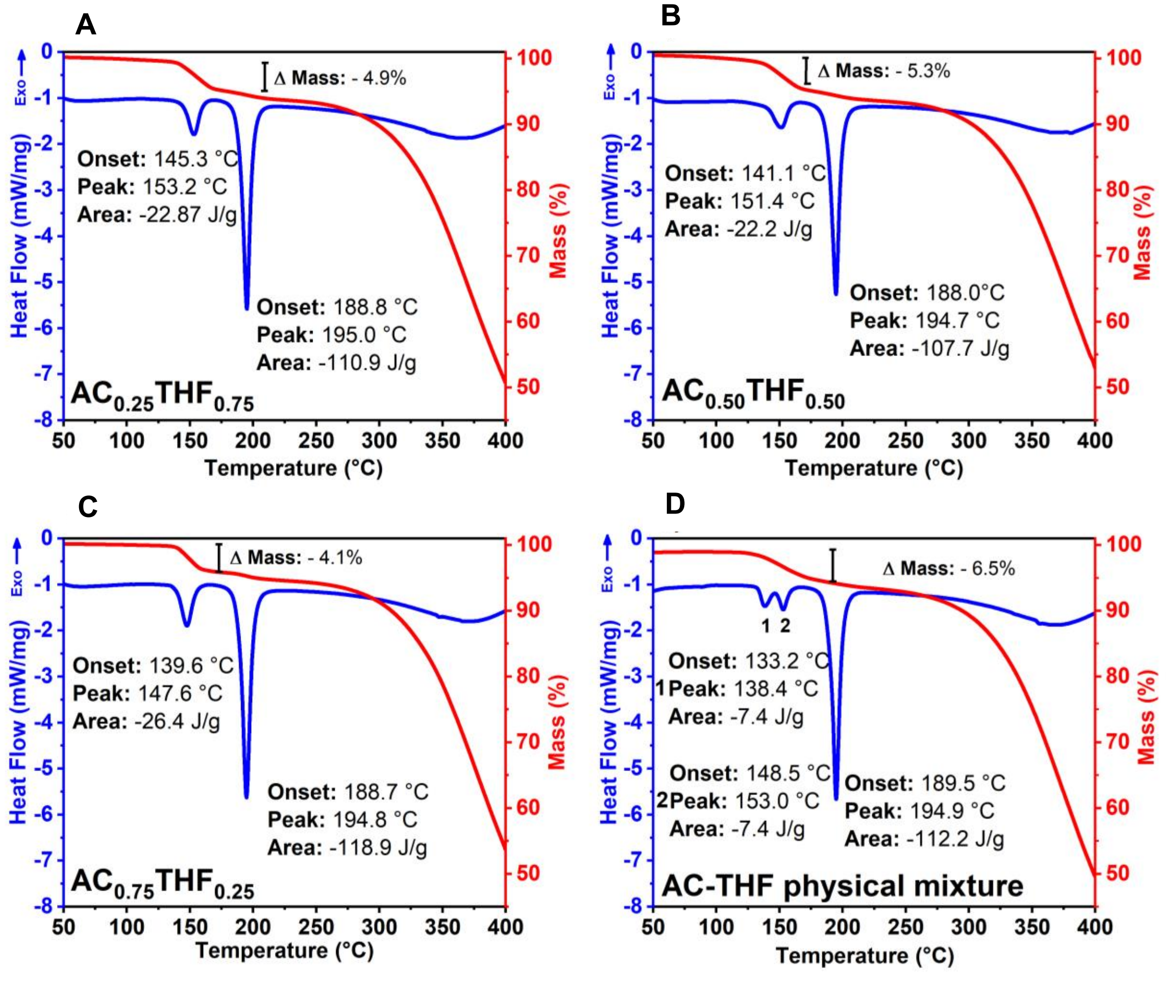


**Figure S8.** DSC (blue line) and TGA (red line) of solvate-solid solutions (nominal composition), of **AC:THF**, A) 0.25:0.75, B) 0.5:0.5, C) 0.75:0.25. D) Physical Mixture.

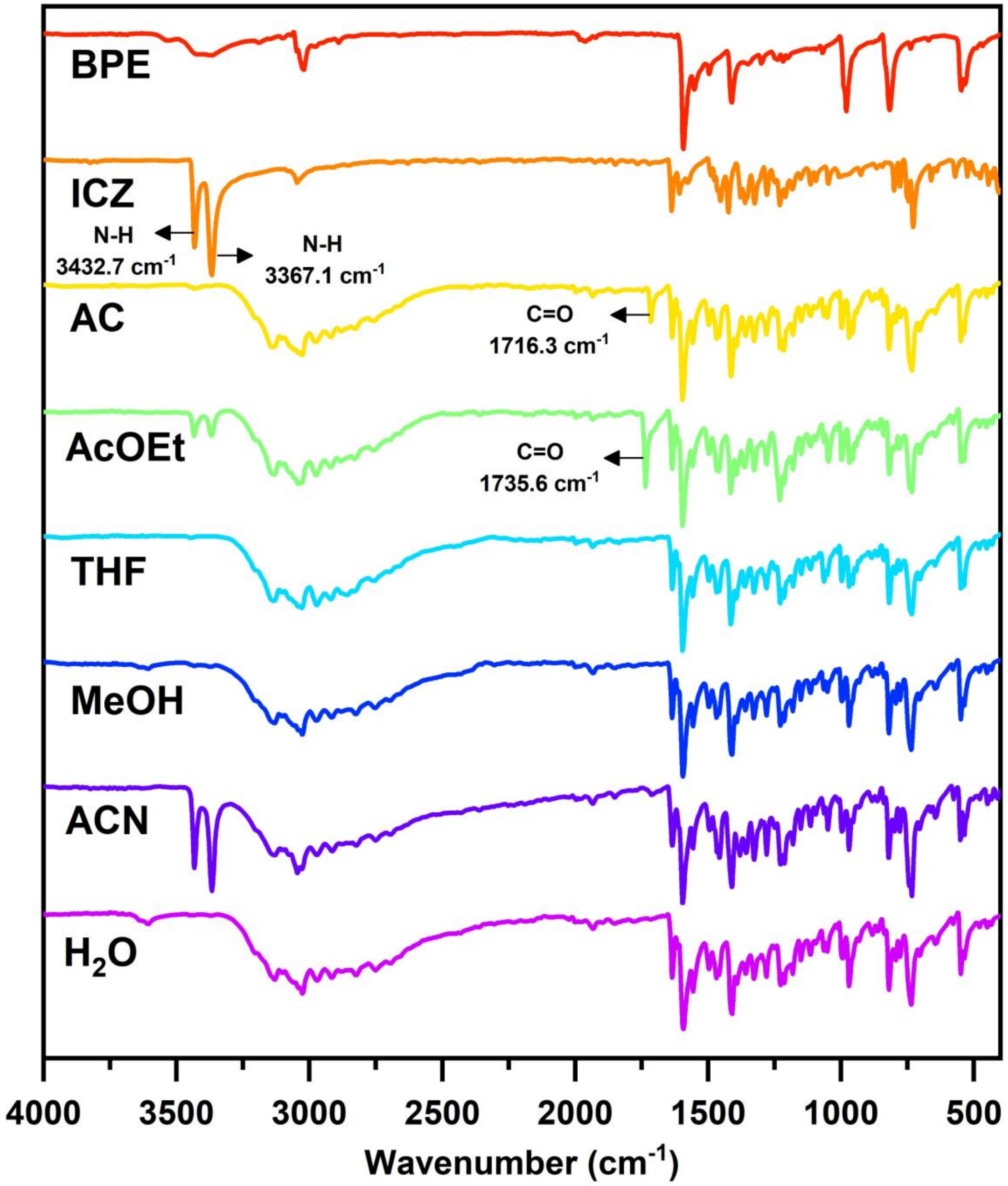


**Figure S9.** FTIR-ATR comparison of the conformers (**ICZ**, **BPE**) and solvate-cocrystals.

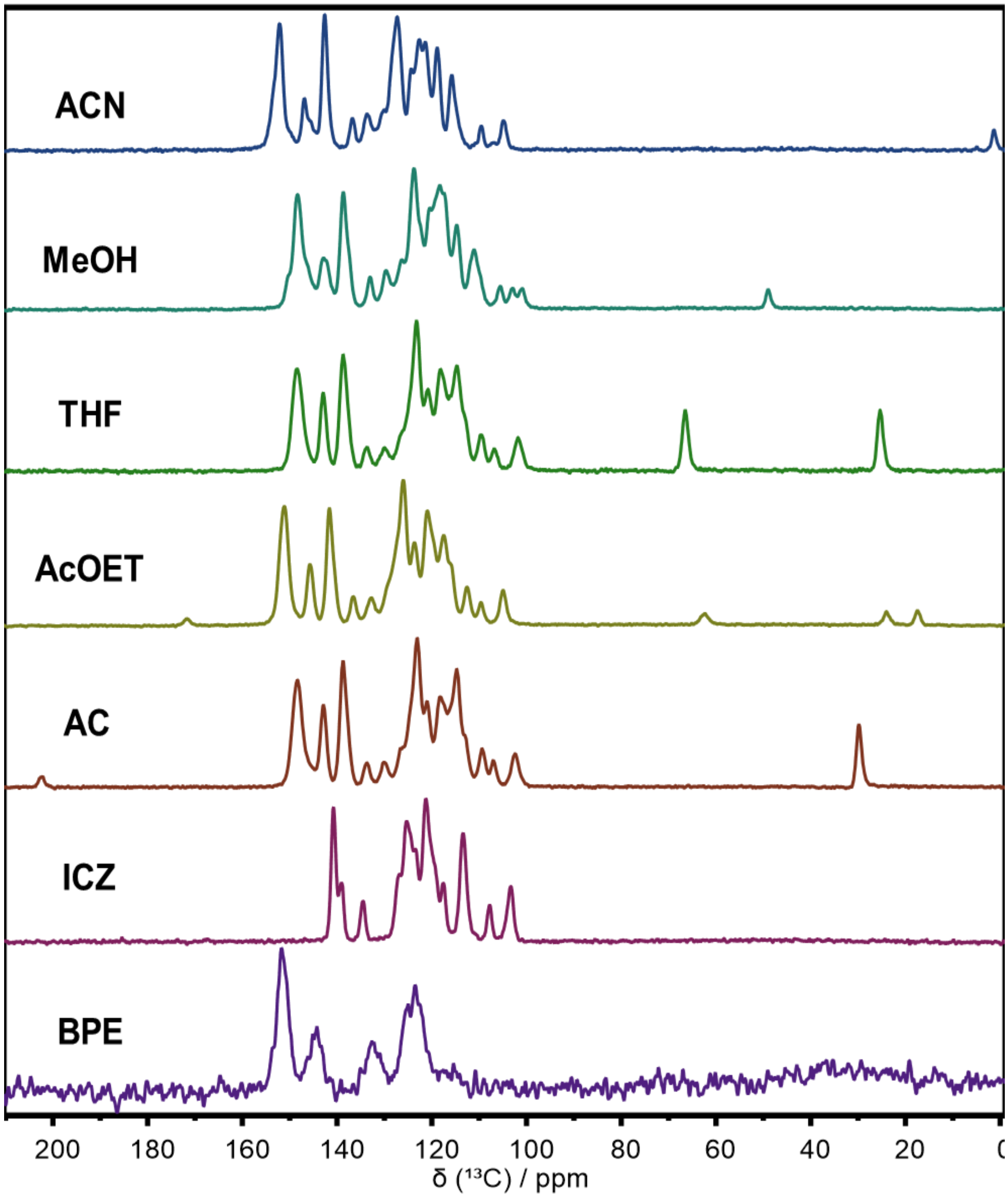


**Figure S10.** $^{13}C$ ss-NMR CP-MAS comparison among starting materials and solvate-cocrystals.

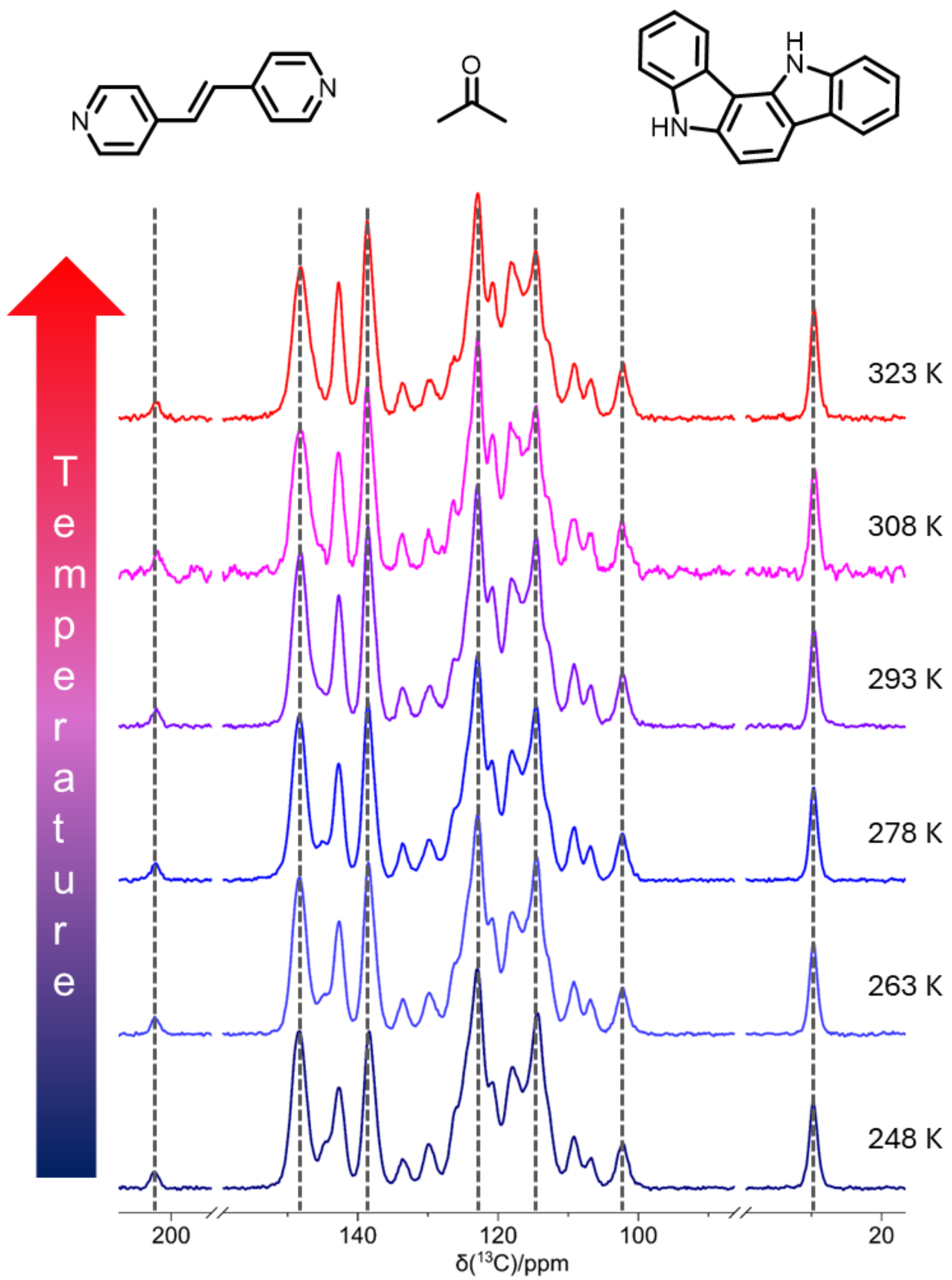


**Figure S11.** Variable temperature $^{13}C$ CP MAS of the solvate AC.

Additionally, the $^{13}C$ CP MAS spectra, recorded between 248 K and 323 K, show that the signals of the cocrystal remain stable, which indicates the stability of the cocrystals and the presence of the solvent within the pore. The intensity of the signals for acetone did not vary with increasing temperature, and the relative intensity between channels didn't change.

**Table S6.** Crystallographic data of thermosalient solvate cocrystals.

| | AC-100 K | AC-298 K | AcOEt-150K | AcOEt-298K | THF-150 K | THF-298 K |
|---|---|---|---|---|---|---|
| **Formula** | 4($C_{18}H_{12}N_2$), 6($C_{12}H_{10}N_2$), 2($C_3H_6O$) | 4($C_{18}H_{12}N_2$), 6($C_{12}H_{10}N_2$), 2($C_3H_6O$) | $C_{18}H_{12}N_2$, 1.5($C_{12}H_{10}N_2$), 0.5($C_4H_8O_2$) | $C_{18}H_{12}N_2$, 1.5($C_{12}H_{10}N_2$), 0.5($C_4H_8O_2$) | 2($C_{18}H_{12}N_2$), 3($C_{12}H_{10}N_2$), $C_4H_8O$ | 2($C_{18}H_{12}N_2$), 3($C_{12}H_{10}N_2$), $C_4H_8O$ |
| **Formula weight** | 2234.65 | 2234.65 | 573.68 | 573.68 | 1131.36 | 1131.35 |
| **Temperature (K)** | 100(2) | 298(2) | 150(2) | 298(2) | 150(2) | 298(2) |
| **System** | Monoclinic | Monoclinic | Monoclinic | Monoclinic | Monoclinic | Monoclinic |
| **Space Group** | C2/c | C2/c | C 2/c | C 2/c | C2/c | C2/c |
| **a (Å)** | 30.4046(9) | 31.1146(9) | 30.9333(8) | 31.5491(10) | 30.6660(7) | 31.4934(16) |
| **b (Å)** | 10.2492(3) | 10.3329(3) | 10.2624(3) | 10.3951(3) | 10.3745(3) | 10.3614(5) |
| **c (Å)** | 18.6914(5) | 18.8118(5) | 18.7531(5) | 18.7299(6) | 18.6477(4) | 18.7524(9) |
| **α (°)** | 90 | 90 | 90 | 90 | 90 | 90 |
| **β (°)** | 98.4490(10) | 98.9520(10) | 98.665(10) | 98.7368(12) | 98.694(1) | 98.967(2) |
| **γ (°)** | 90 | 90 | 90 | 90 | 90 | 90 |
| **Radiation** | MoKα, λ = 0.71073 Å | MoKα, λ = 0.71073 Å | MoKα, λ = 0.71073 Å | MoKα, λ = 0.71073 Å | MoKα, λ = 0.71073 Å | MoKα, λ = 0.71073 Å |
| **ρ ($g/cm^3$)** | 1.288 | 1.242 | 1.295 | 1.255 | 1.281 | 1.243 |
| **V ($Å^3$)** | 5761.5(3) | 5974.4(3) | 5885.2(3) | 6071.3(3) | 5864.5(3) | 6044.4(5) |
| **Z** | 2 | 2 | 8 | 8 | 4 | 4 |
| **Abs coefficient ($mm^{-1}$)** | 0.078 | 0.076 | 0.08 | 0.077 | 0.078 | 0.076 |
| **F(000)** | 2352 | 2352 | 2416 | 2416 | 2384 | 2384 |
| **Crystal size (mm)** | 0.313 x 0.297 x 0.284 | 0.406 x 0.234 x 0.146 | 0.371 x 0.358 x 0.262 | 0.386x0.279x0.272 | 0.327 x 0.175 x 0.111 | 0.379 x 0.258 x 0.144 |
| **Collected reflections** | 95336 | 83947 | 98851 | 58452 | 90880 | 82046 |
| **Independent reflections** | 8402 | 9074 | 8956 | 6172 | 6716 | 9187 |
| **Data/rest/param** | 8402/25/415 | 9074/25/414 | 8956/174/488 | 6172/180/465 | 6716/44/421 | 9187/208/466 |
| **Goodness-of-fit on $F^2$** | 1.078 | 1.040 | 1.059 | 1.114 | 1.04 | 1.078 |
| **Final R indices (I>2s(I))** | R=0.0730 wR=0.1250 | R=0.0524 wR=0.1425 | R=0.0637 wR=0.1277 | R=0.1060 wR=0.2791 | R=0.0575 wR=0.1446 | R=0.0692 wR=0.1712 |
| **R indices (all data)** | R=0.1198 wR=0.1541 | R=0.0704 wR=0.1604 | R=0.0944 wR=0.1502 | R=0.1389 wR=0.3214 | R=0.0726 wR=0.1628 | R=0.0953 wR=0.1969 |
| **CCDC Number** | 2497070 | 2497072 | 2497067 | 2497066 | 2497071 | 2497068 |

**Table S7.** Crystallographic data of solvate cocrystals **MeOH**, **ACN**, **$H_2O$**.

| | **MeOH** | **ACN** | **$H_2O$** |
|---|---|---|---|
| **Formula** | 2($C_{18}H_{12}N_2$), 3.5($C_{12}H_{10}N_2$), $CH_4O$ | 2($C_{18}H_{12}N_2$), 3.5($C_{12}H_{10}N_2$), $C_2H_3N$ | 4($C_{18}H_{12}N_2$), 7($C_{12}H_{10}N_2$), $H_2O$ |
| **Formula weight (g/mol)** | 1182.4 | 1191.41 | 2318.73 |
| **Temperature (K)** | 100(2) | 100(2) | 150(2) |
| **System** | Triclinic | Monoclinic | Triclinic |
| **Space Group** | P-1 | $P2_1/n$ | P-1 |
| **a (Å)** | 9.9093(3) | 19.3432(5) | 9.8721(3) |
| **b (Å)** | 16.4848(5) | 9.9590(2) | 16.7361(6) |
| **c (Å)** | 18.9890(6) | 32.9577(8) | 18.5430(6) |
| **α (°)** | 101.957(10) | 90 | 99.927(1) |
| **β (°)** | 93.054(10) | 105.552(10) | 93.859(1) |
| **γ (°)** | 97.255(10) | 90 | 97.575(1) |
| **Radiation** | MoKα, λ = 0.71073 Å | MoKα, λ = 0.71073 Å | MoKα, λ = 0.71073 Å |
| **ρ ($g/cm^3$)** | 1.309 | 1.294 | 1.293 |
| **V ($Å^3$)** | 3000.21(16) | 6113.5(3) | 2978.74(17) |
| **Z** | 2 | 4 | 1 |
| **Abs coefficient ($mm^{-1}$)** | 0.08 | 0.078 | 0.078 |
| **F(000)** | 1244 | 2504 | 1218 |
| **Crystal size (mm)** | 0.390x0.311x0.282 | 0.406x0.305x0.206 | 0.340x0.268x0.126 |
| **Collected reflections** | 208651 | 146377 | 106137 |
| **Independent reflections** | 19065 | 22233 | 14131 |
| **Data/rest/param** | 19065/606/982 | 22233/922/1034 | 14131/520/975 |
| **Goodness-of-fit on $F^2$** | 1.05 | 1.019 | 1.094 |
| **Final R indices (I>2s(I))** | R=0.0771 wR=0.1483 | R=0.0510 wR=0.1382 | R=0.0836 wR=0.1695 |
| **R indices (all data)** | R=0.1094 wR=0.1694 | R=0.0663 wR=0.1382 | R=0.1463 wR=0.2075 |
| **CCDC Number** | 2497073 | 2497074 | 2497069 |

**Table S8.** Crystallographic data of a solid solution **AC-THF**.

| | **ICZ_BPE_AC_THF** |
|---|---|
| **Formula** | 2($C_{18}H_{12}N_2$), 3($C_{12}H_{10}N_2$), 0.66($C_4H_8O$), 0.34($C_3H_6O$) |
| **Formula weight (g/mol)** | 1126.58 |
| **Temperature (K)** | 223(2) |
| **System** | Monoclinic |
| **Space Group** | C 2/c |
| **a (Å)** | 30.9630(10) |
| **b (Å)** | 10.3406(3) |
| **c (Å)** | 18.7098(6) |
| **α (°)** | 90 |
| **β (°)** | 98.7650(10) |
| **γ (°)** | 90 |
| **Radiation** | MoKα, λ = 0.71073 Å |
| **ρ (g/cm³)** | 1.264 |
| **V (Å³)** | 5920.5(3) |
| **Z** | 4 |
| **Abs coefficient ($mm^{-1}$)** | 0.077 |
| **F(000)** | 2373 |
| **Crystal size (mm)** | 0.282 × 0.312 × 0.364 |
| **Collected reflections** | 164,546 |
| **Independent reflections** | 11,258 |
| **Data/rest/param** | 11,258 / 33 / 412 |
| **Goodness-of-fit on $F^2$** | 1.033 |
| **Final R indices (I > 2σ(I))** | $R_1$ = 0.0624; $wR_2$ = 0.1536 |
| **R indices (all data)** | $R_1$ = 0.0824; $wR_2$ = 0.1788 |
| **CCDC Number** | 2504912 |

**Table S9.** Crystallographic data for the squeezed form.

| | **ICZ_BPE (squeeze)** |
|---|---|
| **Chemical Formula** | $2(C_{18}H_{12}N_2)$, $3(C_{12}H_{10}N_2)$ |
| **Formula Weight** | 1203.513 |
| **Crystal System** | Monoclinic |
| **Space Group** | C2/c |
| **a (Å)** | 30.369(2) |
| **b (Å)** | 10.397(2) |
| **c (Å)** | 18.679(3) |
| **α (°)** | 90 |
| **β (°)** | 98.941(4) |
| **γ (°)** | 90 |
| **Volume ($Å^3$)** | 5826.2(15) |
| **Z** | 4 |
| **Temperature (K)** | 100(1) |
| **Radiation** | Synchrotron |
| **Wavelength (Å)** | 0.6702 |
| **Density ($g/cm^3$)** | 1.372 |
| **Absorption Coefficient ($mm^{-1}$)** | 0.075 |
| **F(000)** | 2545.121 |
| **Reflections Measured** | 46844 |
| **Reflections Independent** | 8362 |
| **R1 ($I>2\sigma(I)$)** | 0.0474 |
| **wR2** | 0.1396 |
| **Goodness of Fit** | 1.0217 |

## Solvate cocrystals with deuterated solvents

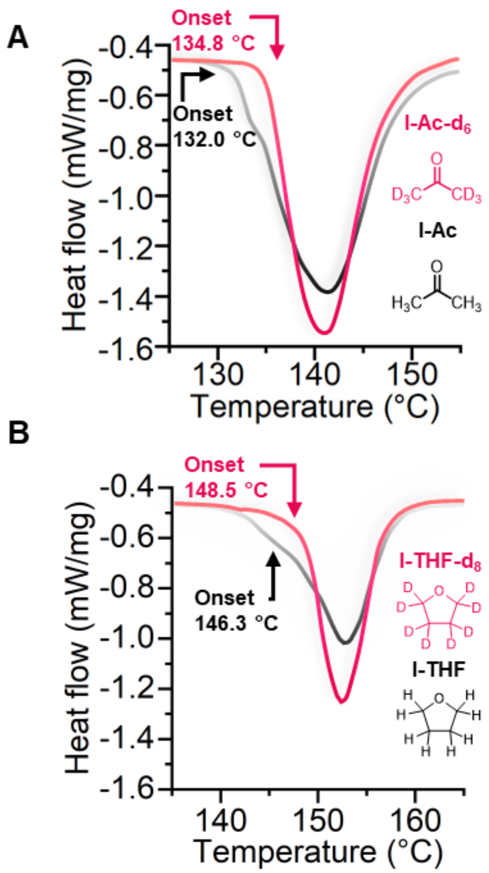


**Figure S12.** Effect of deuteration on the salient effect temperature, A) DSC of **AC-*d*$_6$**, B) DSC of **THF-*d8***.

**Statistics of frequency jump behavior of solvate cocrystals**

**Table S10**. Crystal dimensions employed in the K-means analysis for cocrystal-solvate **AC**.

| Crystal | A (mm) | B (mm) | C (mm) | Jump/Explosion | Cluster K-means |
|---|---|---|---|---|---|
| 1 | 2.93 | 0.68 | 0.47 | No | Small |
| 2 | 2.53 | 0.49 | 0.22 | Yes | Small |
| 3 | 2.68 | 0.85 | 0.61 | Yes | Small |
| 4 | 3.22 | 0.88 | 0.71 | Yes | Small |
| 5 | 3.06 | 0.8 | 0.51 | Yes | Small |
| 6 | 2.44 | 1.3 | 0.71 | Yes | Small |
| 7 | 2.21 | 0.74 | 0.25 | Yes | Small |
| 8 | 3.2 | 0.58 | 0.49 | No | Small |
| 9 | 2.21 | 0.6 | 0.34 | No | Small |
| 10 | 4.67 | 1.62 | 0.77 | Yes | Medium |
| 11 | 3.99 | 1.03 | 0.93 | Yes | Medium |
| 12 | 4.37 | 1.02 | 0.31 | No | Medium |
| 13 | 4.11 | 1.03 | 0.65 | No | Medium |
| 14 | 3.98 | 1.01 | 1.06 | Yes | Medium |
| 15 | 4.36 | 0.94 | 1.74 | Yes | Medium |
| 16 | 3.6 | 1.00 | 0.53 | No | Medium |
| 17 | 4.01 | 1.25 | 0.83 | Yes | Medium |
| 18 | 7.63 | 1.13 | 0.57 | Yes | Large |
| 19 | 6.15 | 2.41 | 1.05 | Yes | Large |
| 20 | 7.89 | 1.47 | 1.06 | Yes | Large |
| 21 | 6.47 | 1.15 | 0.36 | Yes | Large |

**Table S11.** Crystal dimensions employed in the K-means analysis for cocrystal-solvate **AcOEt**.

| Crystal | A (mm) | B (mm) | C (mm) | Jump/Explosion | Cluster K-means |
|---|---|---|---|---|---|
| 1 | 1.93 | 0.39 | 0.35 | No | Small |
| 2 | 2.21 | 0.51 | 0.79 | No | Small |
| 3 | 3.75 | 1.6 | 0.7 | Yes | Medium |
| 4 | 4.68 | 0.77 | 0.53 | Yes | Medium |
| 5 | 4.3 | 1.65 | 0.85 | Yes | Medium |
| 6 | 6.06 | 1.72 | 0.75 | Yes | Large |

| | | | | | |
|---|---|---|---|---|---|
| 7 | 7.37 | 1.87 | 0.72 | Yes | Large |
| 8 | 7.14 | 1.29 | 1.07 | Yes | Large |
| 9 | 6.7 | 1.51 | 1.19 | Yes | Large |
| 10 | 7.51 | 2.03 | 0.81 | Yes | Large |
| 11 | 9.94 | 2.47 | 1.06 | Yes | Large |
| 12 | 7.84 | 2.16 | 0.98 | No | Large |

**Table S12.** Crystal dimensions employed in the K-means analysis for cocrystal-solvate **THF**.

| Sample | A (mm) | B (mm) | C (mm) | Jump/Explosion | Cluster K-means |
|---|---|---|---|---|---|
| 1 | 1.57 | 0.63 | 0.36 | No | Small |
| 2 | 1.75 | 0.62 | 0.33 | No | Small |
| 3 | 1.79 | 0.57 | 0.33 | No | Small |
| 4 | 2.12 | 0.41 | 0.21 | No | Medium |
| 5 | 2.14 | 0.75 | 0.3 | Si | Medium |
| 6 | 2.26 | 0.33 | 0.23 | No | Medium |
| 7 | 2.32 | 0.47 | 0.19 | No | Medium |
| 8 | 3.24 | 0.7 | 0.27 | No | Large |
| 9 | 3.42 | 0.33 | 0.6 | No | Large |
| 10 | 3.91 | 1.02 | 0.7 | No | Large |
| 11 | 3.96 | 0.66 | 0.55 | No | Large |

**Table S13.** Average length, weight, and thickness (A, B, C), and their dispersion for each group.

| | Group | A ± σ (mm) | B ± σ (mm) | C ± σ (mm) |
|---|---|---|---|---|
| **AcOEt** | Small | 2.07 ± 0.20 | 0.45 ± 0.08 | 0.57 ± 0.31 |
| | Medium | 4.24 ± 0.47 | 1.34 ± 0.49 | 0.69 ± 0.16 |
| | Large | 7.51 ± 1.22 | 1.86 ± 0.40 | 0.94 ± 0.18 |
| **AC** | Small | 2.72 ± 0.40 | 0.77 ± 0.24 | 0.48 ± 0.18 |
| | Medium | 4.14 ± 0.33 | 1.11 ± 0.22 | 0.85 ± 0.43 |
| | Large | 7.04 ± 0.85 | 1.54 ± 0.60 | 0.76 ± 0.35 |
| **THF** | Small | 1.70 ± 0.12 | 0.61 ± 0.03 | 0.34 ± 0.02 |
| | Medium | 2.21 ± 0.10 | 0.49 ± 0.18 | 0.23 ± 0.05 |
| | Large | 3.63 ± 0.36 | 0.68 ± 0.28 | 0.53 ± 0.18 |

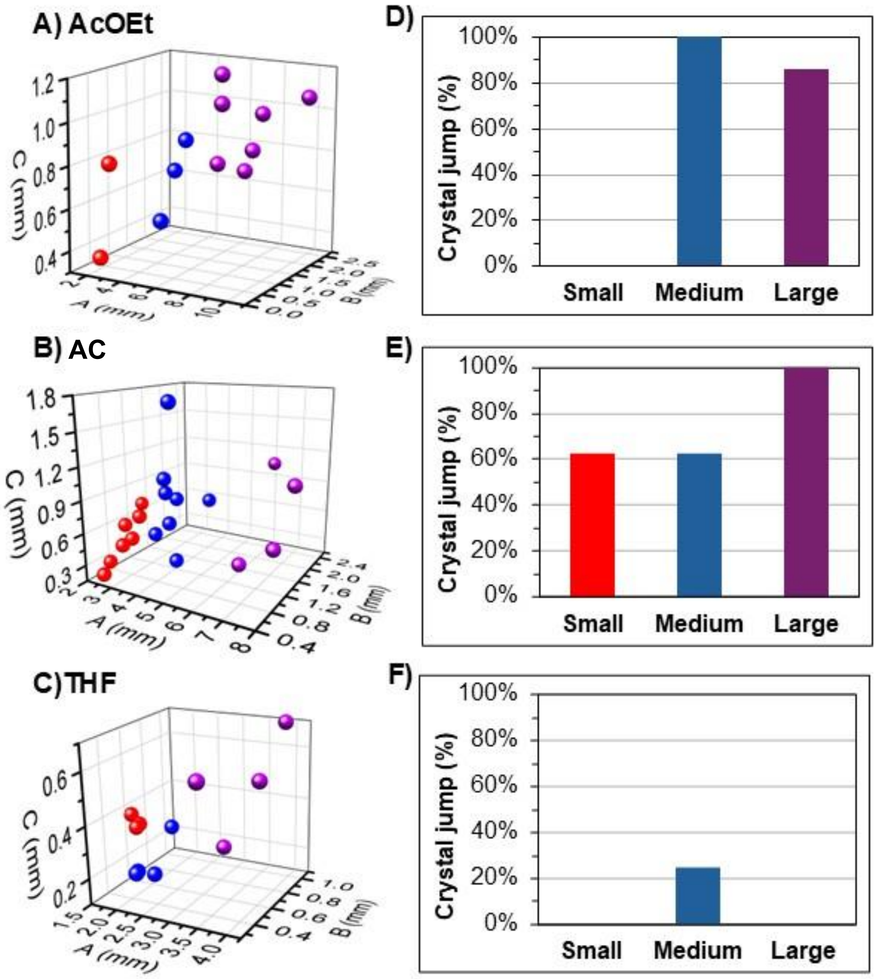


**Figure S13.** Crystal grouping obtained by k-means clustering (K = 3) for crystals grown from A) **AcOEt,** B) **AC**, and C) **THF,** D–F) Jump percentage for each crystal group.

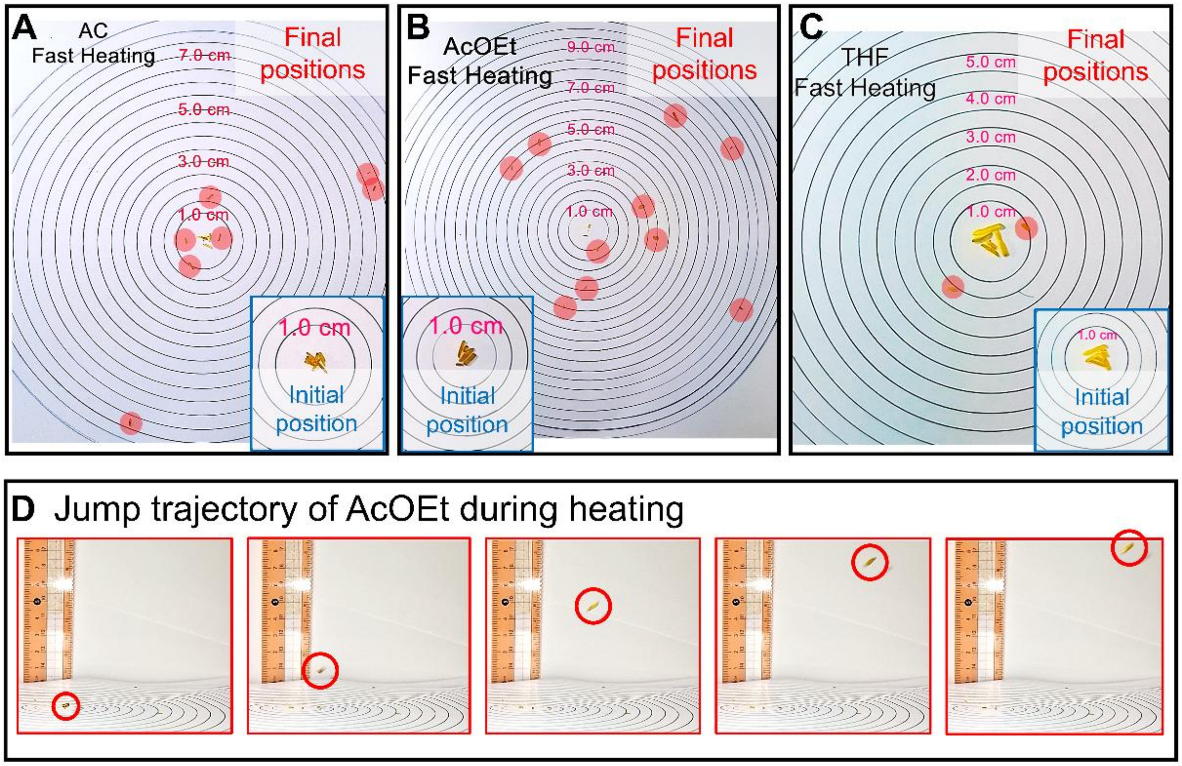


**Figure S14.** Qualitative and illustrative trajectory analysis of the thermosalient response in the three channel-types cocrystal solvates. A) **AC**, B) **AcOEt**, C) **THF**, D) Trajectory of the jump of a single crystal with **AcOEt**. Moreover, the motion of solvate cocrystals frequently does not correspond to the displacement of an intact crystal; video analysis shows that fragments are expelled during desolvation. The displacement of crystal fragments depends on the orientation and size of the crystals, as well as on the contact surface (friction), internal defects, and the mass of the expelled fragments, making it nontrivial to determine the effect of each variable.

## Gas Chromatography coupled to mass spectrometry

In a sealed tube, around 5 mg of each cocrystal was heated to the temperature of the thermosalient effect. After the crystals appeared opaque, we collected a vapor sample with a syringe and immediately analyzed it by gas chromatography coupled to mass spectrometry; a chromatogram was acquired.

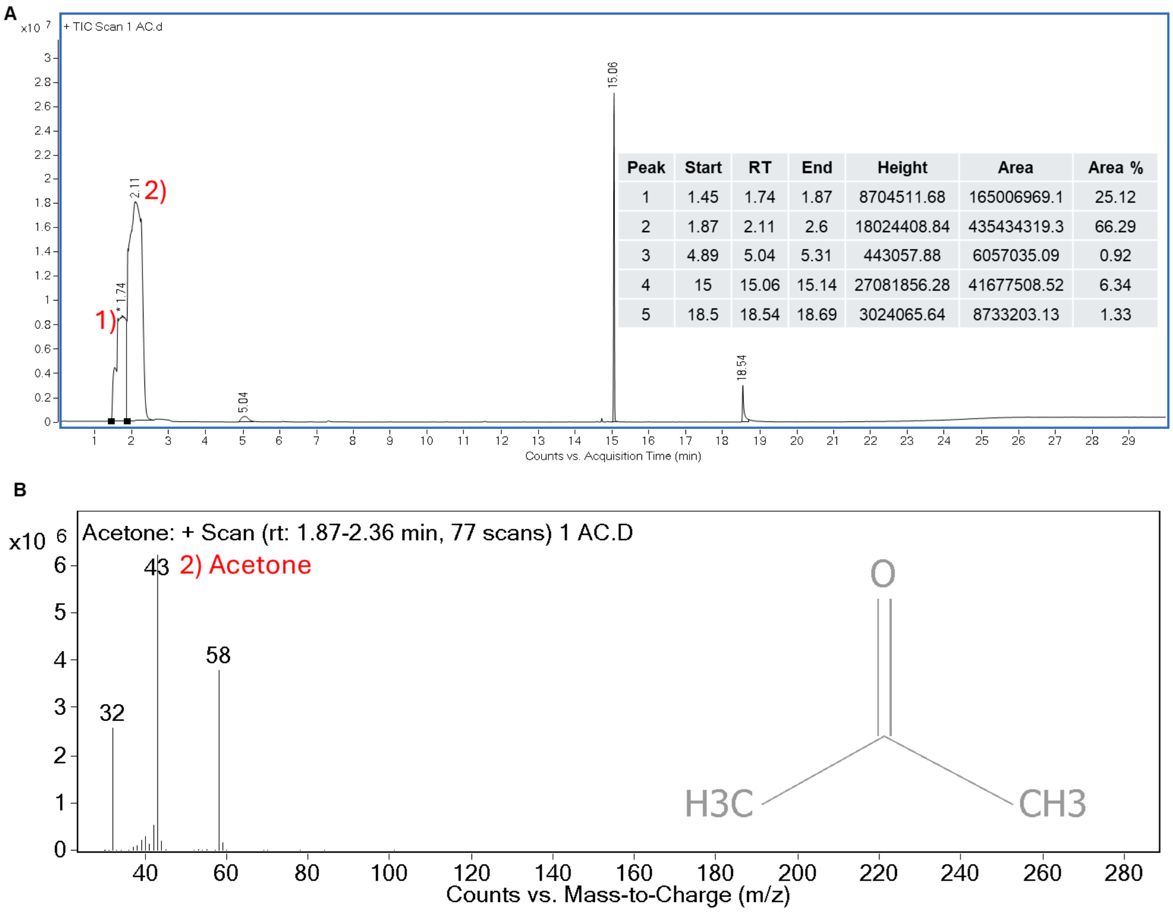


| Peak | Start | RT | End | Height | Area | Area % |
|---|---|---|---|---|---|---|
| 1 | 1.45 | 1.74 | 1.87 | 8704511.68 | 165006969.1 | 25.12 |
| 2 | 1.87 | 2.11 | 2.6 | 18024408.84 | 435434319.3 | 66.29 |
| 3 | 4.89 | 5.04 | 5.31 | 443057.88 | 6057035.09 | 0.92 |
| 4 | 15 | 15.06 | 15.14 | 27081856.28 | 41677508.52 | 6.34 |
| 5 | 18.5 | 18.54 | 18.69 | 3024065.64 | 8733203.13 | 1.33 |

**Figure S15.** A) Gas Chromatography-Mass Spectrometry analysis of gases from the crystal with Ac after fast heating, B) principal detected molecules based on the peak area, peak 1 corresponds to atmospheric air, and peak 2 corresponds to acetone. Peaks 3, 4, and 5 correspond to solvent impurities.

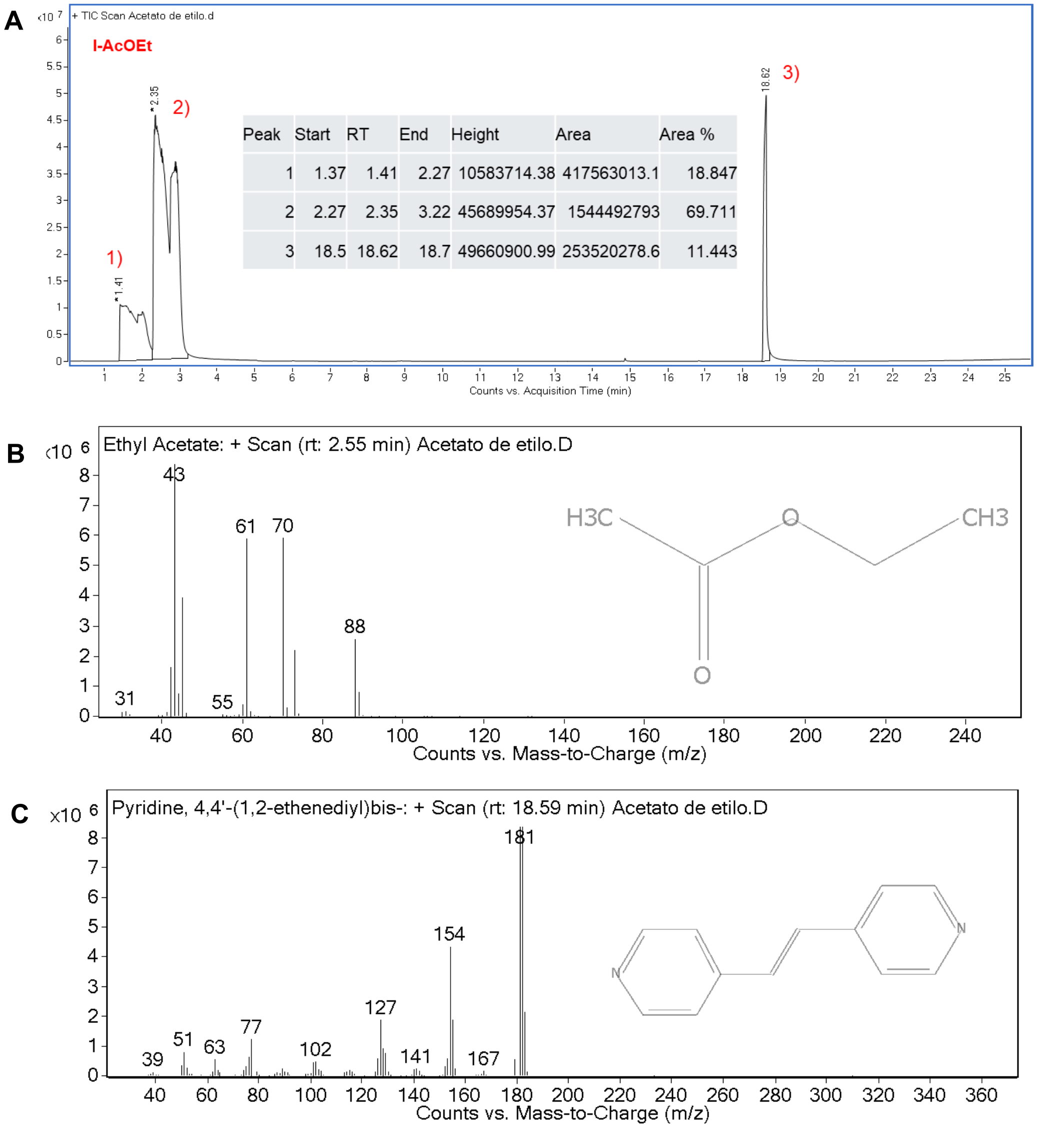


| Peak | Start | RT | End | Height | Area | Area % |
|---|---|---|---|---|---|---|
| 1 | 1.37 | 1.41 | 2.27 | 10583714.38 | 417563013.1 | 18.847 |
| 2 | 2.27 | 2.35 | 3.22 | 45689954.37 | 1544492793 | 69.711 |
| 3 | 18.5 | 18.62 | 18.7 | 49660900.99 | 253520278.6 | 11.443 |

**Figure S16.** Gas Chromatography-Mass Spectrometry analysis of gases from the crystal with **AcOET** after fast heating. Principal detected molecules based on the peak area, peak 1 corresponds to atmospheric air, and peak 2 corresponds to ethyl acetate. C) The peak 3 corresponds to a **BPE**; this sample was heated above 165°C, where the cocrystal starts to sublimate.

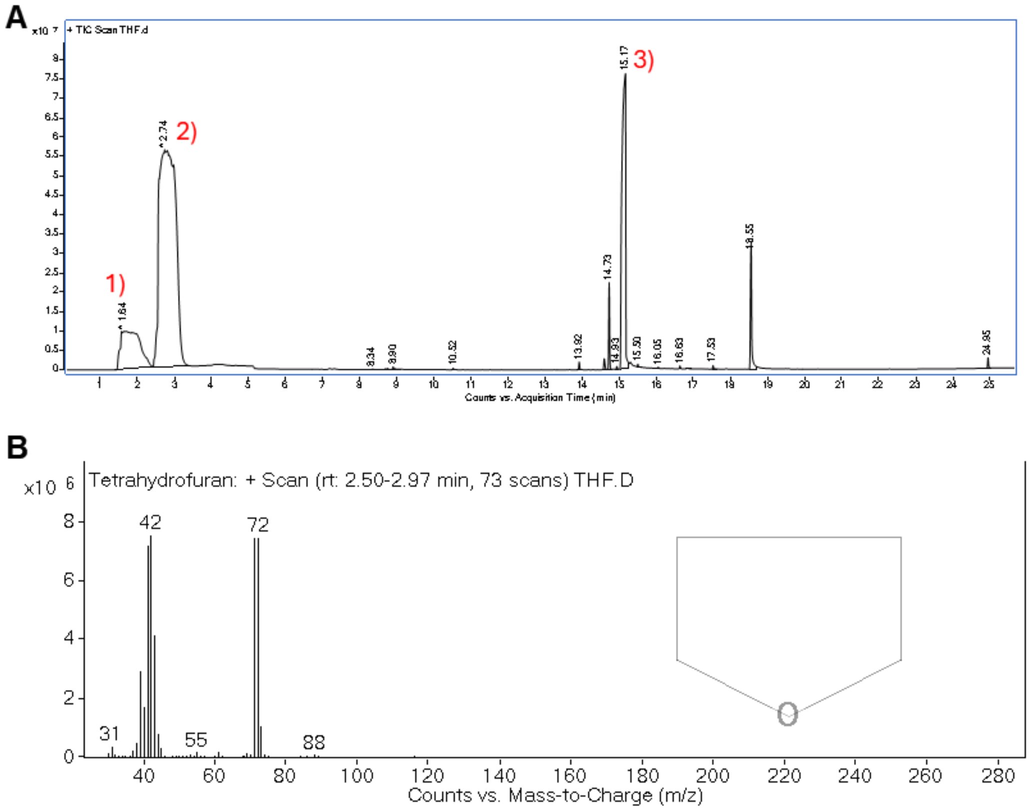


C

| Integration Peak List | | | | | | |
|---|---|---|---|---|---|---|
| Peak | Start | RT | End | Height | Area | Area % |
| 1 | 1.39 | 1.64 | 2.42 | 9733393.39 | 355559510.2 | 12.734 |
| 2 | 2.43 | 2.74 | 3.39 | 56008320.12 | 1768490839 | 63.337 |
| 3 | 15.02 | 15.17 | 15.25 | 76042375.23 | 526009235.1 | 18.839 |

**Figure S17**. Gas Chromatography-Mass Spectrometry analysis of gases from the crystal with **THF** after fast heating. Principal detected molecules based on the peak area, peak 1 corresponds to atmospheric air, and peak 2 corresponds to THF. Also, some THF impurities (peroxides) were detected. C) The peak 3 corresponds to a Butylated hydroxytoluene (BHT), which is present in THF as an antioxidant (Supplier: Sigma Aldrich, BHT concentration 250 ppm).

## Solid solutions.

Cocrystals were prepared using three different solvent mixtures of **AC** and **THF**: 25:75, 50:50, and 75:25 (v/v). Following the optimized method for the pure analogs cocrystals, **ICZ** (50 mg, 0.19 mmol) and **BPE** (55 mg, 0.29 mmol) were placed in a 4 mL vial, and 2 mL of the corresponding solvent mixture was added. The vial was sealed and heated at 85 °C until the solids dissolved. Then, the cap was slightly opened, and the solvent was allowed to evaporate for over 24 hours. The crystals were analyzed by $^1$H NMR in DMSO-$d_6$ to determine the proportion of solvents incorporated into the crystal structure.

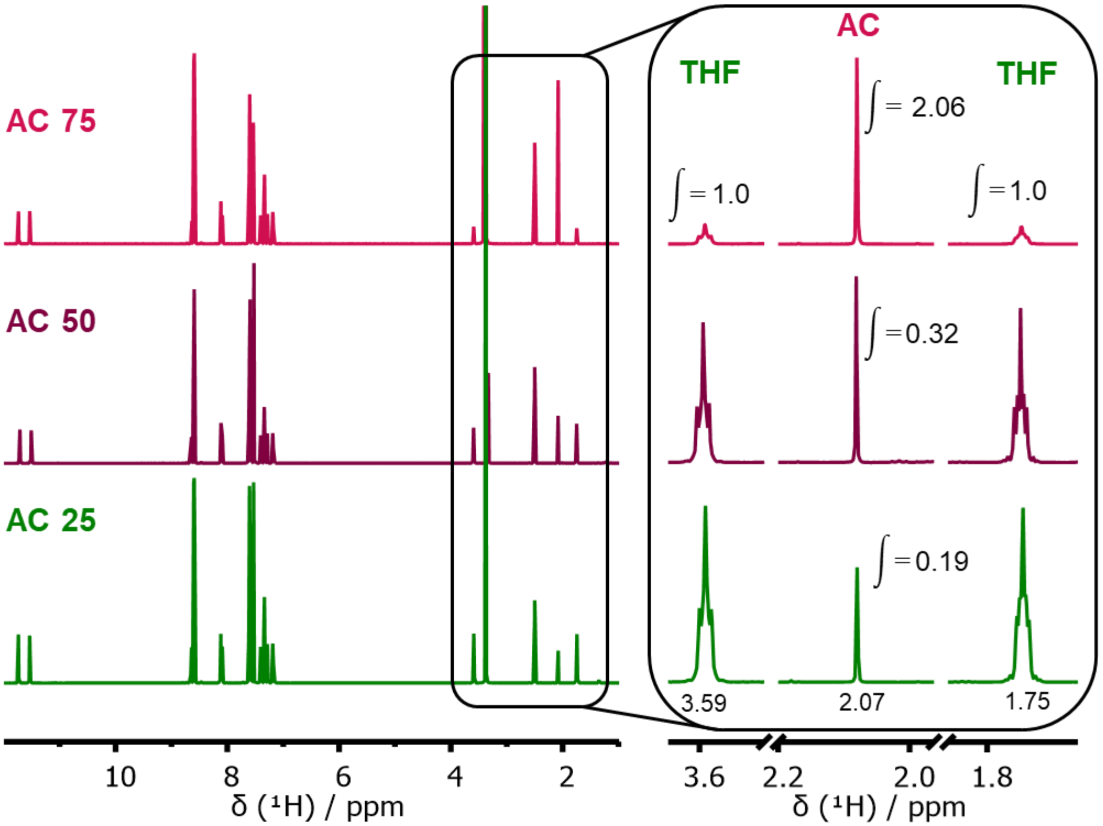


**Figure S18.** $^1$H NMR spectra of solid-solution cocrystals with **AC** and **THF** (700 MHz; DMSO-$d_6$; 293.2 K); the percentages indicate the nominal proportions.

To determine the proportion of each solvent occluded in the cocrystals, 10 mg of freshly obtained material was dissolved in DMSO-$d_6$ and analyzed by $^1$H NMR spectroscopy. The characteristic signals of **THF** were observed at 3.59 and 1.75 ppm, while **AC** -CH3 appeared at 2.07 ppm. The integral of the **THF** signal at 3.59 ppm was set as 1.0, and the corresponding integral value for **AC** was used to

calculate the relative ratio of both solvents. The percentage of each solvent incorporated into the cocrystal was then determined using the following relation.

$$\%_{AC} = \frac{\frac{\int AC}{\#H_{AC}}}{\frac{\int AC}{\#H_{AC}} + \frac{\int THF_{3.59}}{\#H_{THF}}}; \quad \%_{THF} = \frac{\frac{\int THF}{\#H_{THF}}}{\frac{\int AC}{\#H_{AC}} + \frac{\int THF}{\#H_{THF}}}$$

**Equation S1.** Percentage of solvent in the cocrystal.

Where:

$\%_{AC}$: Percentage of **AC**.

$\%_{THF}$: Percentage of **THF**.

$\int AC$: Integral of the signal of **AC**.

$\int THF$: Integral of the signal of **THF** at 3.59 ppm.

$\#H_{AC}$: Number of hydrogens in the acetone molecule.

$\#H_{THF}$: Number of hydrogens in the THF molecule.

**Table S14.** Nominal and corrected proportion of solvents occluded in cocrystals.

| Nominal AC:THF | Acetone (mL) | THF (mL) | Corrected AC:THF |
|---|---|---|---|
| 75:25 | 1.5 | 0.5 | 58:42 |
| 50:50 | 1.0 | 1.0 | 18:82 |
| 25:75 | 0.5 | 1.5 | 11:89 |

These experiments were performed in duplicate and yielded similar results, indicating that the solvent ratio within the cocrystals is reproducible under the tested conditions.

**Variable temperature SCXRD**

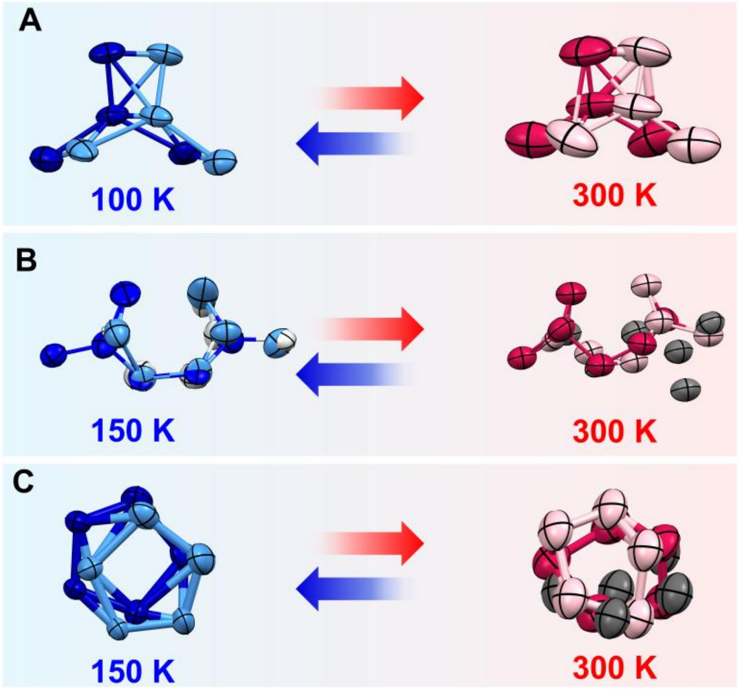


**Figure S19.** Variable temperature SCXRD and the effect of temperature on the temperature to crystal disorder of A) **AC**, B) **I-AcOEt**, and C) **I-THF.**

## $^2$H Solid-state NMR Simulations

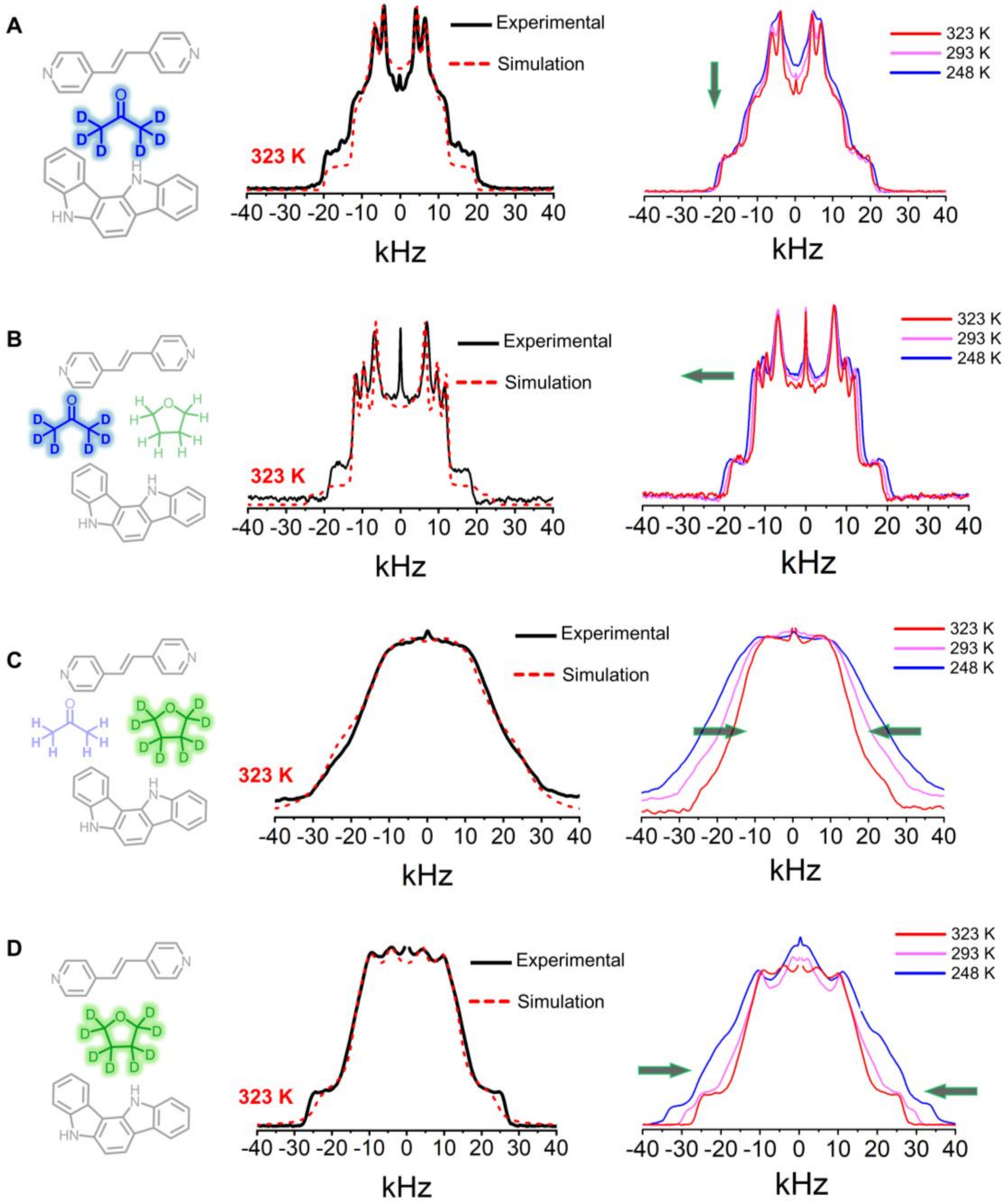


**Figure S20.** $^2$H ss-NMR spectra experimental and simulation at 323 K (Right) and variable temperature spectra for 248K-323 K, for A) **AC**, B) **AC-*d*$_6$**: **THF**, C) **AC:THF-*d*$_8$**, D)**THF-*d*$_8$**.

**Table S15.** Complete parameters for simulation of quadrupolar $^2$H spin-echo ss-NMR for deuterated samples of crystals with **AC-*d*$_6$** at 293 K (20 °C).

| | | **Sample: ·AC-d$_6$ at 293K** | | |
|---|---|---|---|---|
| **Mode** | | 3-fold | 3-fold | 2-fold |
| **Contribution** | | 0.133 | 0.067 | 0.80 |
| **Tensor Settings** | spin | 1 | 1 | 1 |
| | coupling $\delta_p$ | 40 kHz | 40 kHz | 40 kHz |
| | asymmetry $\eta_0$ | 0.0 | 0.0 | 0.0 |
| | tensor relative to | cone frame | cone frame | cone frame |
| **Cone Settings** | timescale of motion | fast limit | fast limit | fast limit |
| | type of motion | 3 sites | 3 sites | 2 sites |
| | cone angle $\theta$ | 66° | 70° | 58° |
| | jump angle $\phi$ | 120° | 120° | 180° |
| | populations | 0.333, 0.333, 0.333 | 0.333, 0.333, 0.333 | 0.5, 0.5 |

**Table S16.** Complete parameters for simulation of quadrupolar $^2$H spin-echo ss-NMR for deuterated samples of crystal with **AC-d$_6$-THF (**50:50) 248 K (-25º C) and 323 K (50°C).

| | | **Sample: AC-*d*$_6$-THF (50:50) at 248 K (-25º C)** | | |
|---|---|---|---|---|
| **Mode** | | 3-fold | 3-fold | 3-fold |
| **Contribution** | | 0.299 | 0.067 | 0.80 |
| **Tensor Settings** | spin | 1 | 1 | 1 |
| | coupling $\delta_p$ | 40 kHz | 40 kHz | 40 kHz |
| | asymmetry $\eta_0$ | 0.0 | 0.46 | 0.72 |
| | tensor relative to | cone frame | cone frame | cone frame |
| **Cone Settings** | timescale of motion | fast limit | fast limit | fast limit |
| | type of motion | 3 sites | 3 sites | 2 sites |
| | cone angle $\theta$ | 70° | 70° | 70° |
| | jump angle $\phi$ | 120° | 120° | 180° |
| | populations | 0.333, 0.333, 0.333 | 0.333, 0.333, 0.333 | 0.5, 0.5 |
| | | **Sample: AC-*d*$_6$-THF (50:50) At 248 K (50º C)** | | |
| **Mode** | | 3-fold | 3-fold | 3-fold |
| **Contribution** | | 0.299 | 0.067 | 0.80 |
| **Tensor Settings** | spin | 1 | 1 | 1 |
| | coupling $\delta_p$ | 40 kHz | 40 kHz | 40 kHz |
| | asymmetry $\eta_0$ | 0.0 | 0.4 | 0.65 |
| | tensor relative to | cone frame | cone frame | cone frame |
| **Cone Settings** | timescale of motion | fast limit | fast limit | fast limit |
| | type of motion | 3 sites | 3 sites | 2 sites |
| | cone angle $\theta$ | 70° | 70° | 70° |
| | jump angle $\phi$ | 120° | 120° | 180° |
| | populations | 0.333, 0.333, 0.333 | 0.333, 0.333, 0.333 | 0.5, 0.5 |

**Table S17.** Complete parameters for simulation of quadrupolar $^2$H spin-echo ss-NMR for deuterated samples **THF*d₈*** -20º, 20º, 50º C.

| | | Sample: AC·THF$d_8$ |
|---|---|---|
| **Mode** | | 4-fold |
| **Contribution** | | |
| **Tensor Settings** | spin | 1 |
| | coupling $\delta_p$ | 130 kHz |
| | asymmetry $\eta_0$ | 0.0 |
| | tensor relative to | cone frame |
| **Cone Settings** | timescale of motion | fast limit |
| | type of motion | 4 sites |
| | cone angle $\theta$ | 63° |
| | jump angle $\phi$ | 90° |
| | populations | 0.26, 0.24, 0.26, 0.24 |

**Table S18.** Complete parameters for simulation of quadrupolar $^2$H spin-echo ss-NMR for deuterated samples **AC·THF$d_8$ -20º, 20º, 50º C**

| | | **Sample: AC·THF$d_8$ - 25º C** | **Sample: AC·THF$d_8$ 20º C** | **Sample: AC·THF$d_8$ 50º C** |
|---|---|---|---|---|
| **Mode** | | 4-fold | 4-fold | 4-fold |
| **Tensor Settings** | spin | 1 | 1 | 1 |
| **Tensor Settings** | coupling $\delta_p$ | 130 kHz | 130 kHz | 130 kHz |
| | asymmetry $\eta_0$ | 0.0 | 0.0 | 0.0 |
| | tensor relative to | cone frame | cone frame | cone frame |
| | timescale of motion | fast limit | fast limit | fast limit |
| **Cone Settings** | type of motion | 4 sites | 4 sites | 4 sites |
| | cone angle $\theta$ | 63° | 63° | 63° |
| | jump angle $\phi$ with full width 2σ | 90º 40º | 90º 30º | 90º 12º |
| | populations | 0.25, 0.25, 0.25, 0.25 | 0.25, 0.25, 0.25, 0.25 | 0.26, 0.24, 0.26, 0.24 |

## Appendix I: Characterization of the desolvated (DE) form.

The nature of the desolvated form (**DE**) was characterized first by DSC-TGA; this form is the result of solvent expulsion from the lattice of **AC**, **THF**, and **AcOEt**. In the three cases, the DSC profile is very similar: after the salient event, this form is thermally stable, with no phase transition until the melting point at 189-191°C, where a sharp endothermic process occurs, indicating that the remaining solid is crystalline.

All our attempts to obtain a single crystal of the free-solvent form ended in solvate-cocrystal forms, which this system can be cataloged as a promiscuous solvate. Nevertheless, **DE** was studied using ss-NMR $^{13}C$ CP-MAS. The sample was prepared by heating the cocrystal **AcOEt** (c.a. 70 mg) to 150 °C for 30 minutes.

The diffractogram (Figure S14a) of DE form confirms that the solid is still crystalline; however, the comparison with the solvated form reflects notable differences between them (Figure S6). In the ss-NMR $^{13}C$ CPMAS, the absence of the signals corresponds to the methyl and carbonil groups after heating, demonstrating full evacuation of the solvent. Moreover, the $^{13}C$ CP-MAS shows minor changes in the structure, indicating that DE maintains a similar arrangement with solvated forms (Figure S15), which is expected due to the strong π-π interactions and hydrogen bonds.

More precisely, we hypothesize that the **DE** form could be like the **$H_2O$** form, as suggested by the comparison of theoretical PXRD and experimental results (Figure S21a). In **$H_2O$**, the water molecules are contained within cavities rather than channels. Moreover, compared with all the solvated forms, **$H_2O$** contains 0.5 molecules by asymmetric unit Z', which is the lowest proportion of solvent among all solvated cocrystals, resulting in a more packed structure. We conclude that after heating, the channels partially collapsed, forming discrete cavities (closed channel); the continuity is interrupted by one molecule of **BPE** that blocks the channel (Figure S21b).

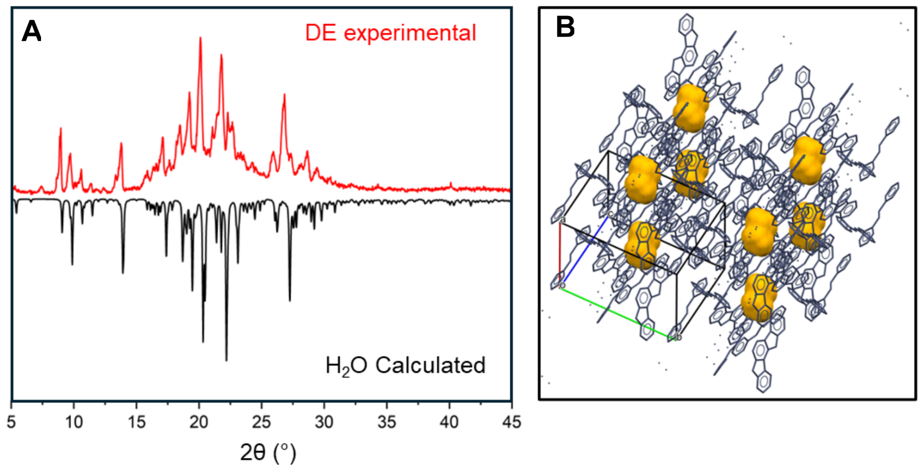


**Figure S21**. Similarities with DE form and **$H_2O$** solvate: A) PXRD comparison, B) cavities in **$H_2O$** solvate.

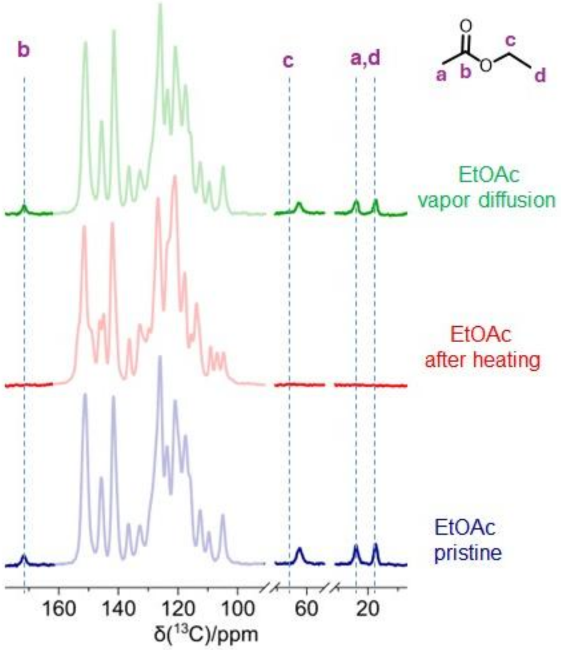


**Figure S22.** ss-NMR $^{13}C$ CP-MAS of reversibility with AcOEt vapors.

## References

(S1) Jian, M.; Song, Z.; Chen, X.; Zhao, J.; Xu, B.; Chi, Z. Afterglows from the Indolocarbazole Families. *Chem. Eng. J.* **2022**, *429*, 132346.
